# Clonal hematopoiesis is associated with distinct rheumatoid arthritis phenotypes

**DOI:** 10.1126/sciadv.adt9846

**Published:** 2025-04-30

**Authors:** Emil Hiitola, Juuso Korhonen, Heidi Kokkonen, Jukka Koskela, Matti Kankainen, Milla Alakuijala, Aoxing Liu, Sofie Lundgren, Paavo Häppölä, Henrikki Almusa, Pekka Ellonen, Paula Savola, Tiina Kelkka, Marjatta Leirisalo-Repo, Riitta Koivuniemi, Ritva Peltomaa, Laura Pirilä, Pia Isomäki, Markku Kauppi, Oili Kaipiainen-Seppänen, Inna Starskaia, Anniina T. Virtanen, Riitta Lahesmaa, Olli Silvennoinen, FinnGen, Giulio Genovese, Andrea Ganna, Solbritt Rantapää-Dahlqvist, Satu Mustjoki, Mikko Myllymäki

**Affiliations:** ^1^ Hematology Research Unit Helsinki, University of Helsinki and Helsinki University Hospital Comprehensive Cancer Center, Helsinki, Finland.; ^2^ Translational Immunology Research program, University of Helsinki, Helsinki, Finland.; ^3^ iCAN Digital Precision Cancer Medicine Flagship, Helsinki, Finland.; ^4^ Department of Public Health and Clinical Medicine/Rheumatology, Umeå University, Umeå, Sweden.; ^5^ Institute for Molecular Medicine Finland, University of Helsinki, Helsinki, Finland.; ^6^ Analytic and Translational Genetics Unit, Massachusetts General Hospital, Boston, MA, USA.; ^7^ Center for Genomic Medicine, Massachusetts General Hospital, Boston, MA, USA.; ^8^ Program in Medical and Population Genetics, Broad Institute of MIT and Harvard, Cambridge, MA, USA.; ^9^ Stanley Center for Psychiatric Research, Broad Institute of MIT and Harvard, Cambridge, MA, USA.; ^10^ Department of Clinical Chemistry and Hematology, HUS Diagnostic Center, Helsinki University Hospital and University of Helsinki, Helsinki, Finland.; ^11^ Inflammation Center, Department of Rheumatology, Helsinki University Hospital and University of Helsinki, Helsinki, Finland.; ^12^ Centre for Rheumatology and Clinical Immunology, Division of Medicine, Turku University Hospital and University of Turku, Turku, Finland.; ^13^ Centre for Rheumatic Diseases, Tampere University Hospital, Tampere, Finland.; ^14^ Faculty of Medicine and Health Technology, Tampere University, Tampere, Finland.; ^15^ Päijät-Häme Central Hospital, Lahti, Finland.; ^16^ Clinicum, Faculty of Medicine, University of Helsinki, Helsinki, Finland.; ^17^ Kuopio University Hospital, Kuopio, Finland.; ^18^ Turku Bioscience Centre, University of Turku and Åbo Akademi University, Turku, Finland.; ^19^ InFLAMES Research Flagship Center, University of Turku, Turku, Finland.; ^20^ Institute of Biotechnology, HiLIFE Helsinki Institute of Life Science, University of Helsinki, Helsinki, Finland.; ^21^ Institute of Biomedicine, University of Turku, Turku, Finland.; ^22^ FIMLAB Laboratories, Tampere, Finland.; ^23^ Stanley Center, Broad Institute of Harvard and MIT, Cambridge, MA, USA.; ^24^ Department of Genetics, Harvard Medical School, Boston, MA, USA.

## Abstract

Clonal hematopoiesis (CH) becomes more prevalent with aging and may influence inflammatory diseases by altering immune function. While CH of indeterminate potential (CHIP) promotes inflammation in nonmalignant conditions, its relationship with rheumatoid arthritis (RA) remains unknown. We analyzed CHIP mutations in RA using two population-level cohorts and patients with newly diagnosed RA. CHIP was associated with prevalent RA in 10,089 FINRISK study participants with whole-exome sequencing (OR, 2.06; *P* = 0.029) and in the FinnGen cohort (*n* = 520,210; OR, 1.49; *P* < 0.001) using single-nucleotide polymorphism array–based CHIP annotation. In FinnGen, CHIP was also associated with inferior overall survival in participants with RA (*P* = 0.013). In newly diagnosed RA (*n* = 573), *DNMT3A*-mutated seropositive patients had increased inflammatory markers and disease activity compared with patients without CHIP. In contrast, *TET2* mutations were enriched in seronegative RA (*P* = 0.009). Our findings provide further evidence for the context-dependent association between CHIP and inflammation, with potential therapeutic implications.

## Teaser

Clonal hematopoiesis mutations are enriched in rheumatoid arthritis and associated with subtype-specific disease phenotypes.

## INTRODUCTION

The role of aging-related clonal hematopoiesis (CH) in inflammatory diseases remains to be fully characterized (*1*, *2*). CH of indeterminate potential (CHIP) is defined by the presence of hematopoietic stem cell (HSC) clones carrying somatic mutations in genes recurrently mutated in myeloid neoplasms (*3*). CHIP predisposes to hematologic malignancies (*3*), but the increased risk of death among CHIP carriers (*4*) is mainly caused by nonmalignant inflammatory phenotypes, such as cardiovascular diseases (CVDs) (*5*). CHIP can promote inflammation via multiple mechanisms, such as via hyperactivation of the NLR family pyrin domain–containing 3 inflammasome and subsequent cytokine release from monocytes/macrophages (*6*). Consequently, CHIP has been implicated in the pathogenesis of inflammatory diseases such as gout (*7*), chronic liver disease (*8*), acute kidney injury (*9*), giant cell arteritis (*10*), and CVD (*4*, *11*). On the other hand, chronic inflammation can boost the expansion of CHIP clones in the bone marrow (*12*, *13*). Anti-inflammatory therapies have shown preliminary efficacy in CVD prevention in high-risk patients with CHIP (*14*), suggesting that identifying CHIP carriers may result in personalized therapeutic opportunities in inflammation-driven disease contexts. In addition to CHIP, other CH subtypes include mosaic chromosomal alterations (mCAs) (*15*, *16*) and CH characterized by somatic variants in lymphoid driver genes (*17*, *18*). Both mCAs (*19*) and lymphoid driver variants (*17*) increase the risk of hematological cancers, but their impact on other disease phenotypes remains to be explored.

Rheumatoid arthritis (RA), one of the most common autoimmune disease primarily affecting joints (*20*), significantly co-occurs with chronic myeloid malignancies (*21*, *22*). Seropositive RA is defined by the presence of anti-cyclic citrullinated peptide antibodies (ACPA) and/or rheumatoid factor (RF), whereas seronegative RA is a more heterogeneous inflammatory disease entity involving patients with clinical RA manifestations in the absence of ACPA and RF (*23*). Clonal expansions of the immune cells have been reported in RA. For example, patients with Felty syndrome, a disease entity characterized by neutropenia, splenomegaly, and seropositive RA, have a high prevalence of *STAT3* mutations in peripheral blood (*24*). CHIP has also been associated with autoimmune diseases in 200 patients who underwent hip arthroplasty for osteoarthritis (*25*) and with RA in a study of 1794 participants aged 80 or older (*26*). However, these preliminary observations have yet to be validated in population-level cohorts, and studies on the prevalence of CHIP in clinically annotated patients with RA have, so far, been limited to a small patient cohort of 59 patients with RA (*27*). Understanding the association between CHIP and RA may provide additional insights into the pathophysiology, prevention, and clinical management of autoimmune diseases.

In this study, we comprehensively analyzed whether CH subtypes are associated with RA in the population level using whole-exome sequencing (WES) data in the FINRISK study (*28*) and single-nucleotide polymorphism (SNP) array data in the FinnGen cohort (*29*). We also evaluated CHIP mutations using next-generation sequencing (NGS) in a cohort of 573 untreated and 59 previously published (*27*) patients with RA as well as 163 healthy controls to elucidate the prevalence of CHIP in distinct RA phenotypes (table S1). Collectively, our approaches highlight the spectrum of CH associated with RA in a context-dependent manner.

## RESULTS

### CH in the FINRISK cohort

To evaluate the association between CH and RA, we reanalyzed WES (*30*) and SNP array data to detect CHIP and mCAs, respectively, in the FINRISK cohort of 10,129 participants that are highly representative of the Finnish population (*30*). The median age of FINRISK participants with WES available was 49 years (range, 25 to 74), and 53% of the participants were male (fig. S1A). The median registry follow-up time in the FINRISK participants was 15.8 years after DNA sampling (range, 0 to 25.9 years).

**Fig. 1.**
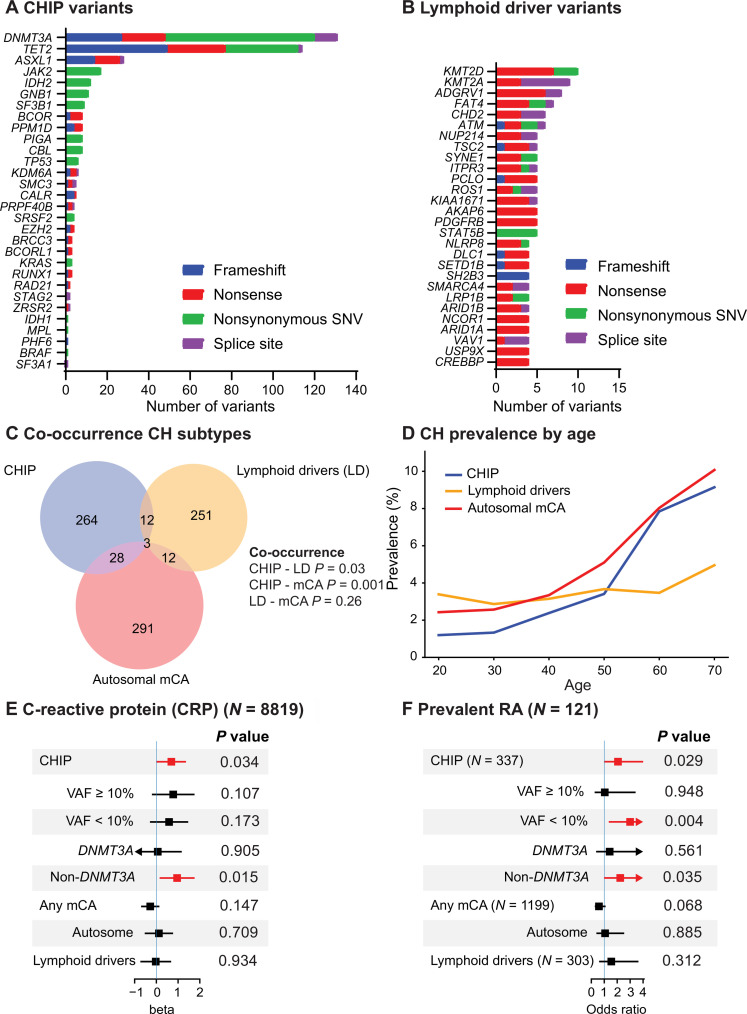
Spectrum of CH and association with RA in the FINRISK study. (**A**) Distribution of CHIP variants by gene and variant type in the FINRISK study. (**B**) Number of variants by variant type in putative lymphoid driver genes in FINRISK. Genes with at least four variants shown in the figure. The full list of variants can be found in table S3. (**C**) Co-occurrence of CH subtypes. *P* values were calculated using Fisher’s exact test. (**D**) Prevalence of CH subtypes in FINRISK participants by age in decades. (**E**) Multivariable models for associations between CH subtypes and CRP. (**F**) Multivariable models for associations between CH subtypes and prevalent RA diagnosis. (E) and (F) are adjusted for age, sex, smoking history (never/ever), and 10 principal components of genetic ancestry. Participants with prevalent hematological cancers were excluded from the analyses of (C) to (F). See figs. S2 and S3 for additional characteristics of CHIP and lymphoid driver variants, respectively.

Among the 10,089 FINRISK participants with WES data (Materials and Methods), the median exome target coverage was 48× (fig. S1C). We identified a total of 411 variants in CHIP genes in 381 FINRISK participants (tables S2 and S3 and fig. S2). Among those with CHIP, most participants (352 of 381, 92%) had one CHIP variant (fig. S2G). Consistent with prior reports (*31*, *32*), the most recurrently mutated genes included *DNMT3A*, *TET2*, and *ASXL1* (Fig. 1A). The ratio of *DNMT3A* variants to *TET2* variants was lower in our cohort compared with that in UK Biobank (UKBB) (*31*) (1.15 versus 2.37, respectively), largely driven by differences in target coverages (fig. S2H). Therefore, the differences in the relative prevalence of *DNMT3A* and *TET2* variants were likely caused by different exome capture kits used in the studies (*30*, *31*). Most of the mutations were missense (fig. S2A), and the most common nucleotide substitution was cytosine to thymine (C>T) (fig. S2B). The association between CHIP variants and sex was also consistent with the UKBB data (fig. S2E) (*31*). The distribution of variant allele frequencies (VAFs) by mutated genes and variant consequences is outlined in fig. S2 (C and D). Only two variants had VAF of below 2%, consistent with 2% being the threshold for CHIP detection in WES data (*3*). Notably, we observed no somatic hotspot variants in *UBA1*, a gene underlying VEXAS syndrome characterized by autoimmune phenotypes (*33*, *34*) as expected based on recently reported population prevalence of *UBA1* mutations in the United States (*35*).

We also evaluated the spectrum of lymphoid driver variants (*17*, *36*) in the FINRISK cohort (Materials and Methods, table S4, and Fig. 1B). We identified 354 variants in 346 participants in lymphoid driver genes (table S3 and fig. S3). The most recurrently mutated lymphoid driver genes included *KMT2D*, *KMT2A*, and *ADGRV1*; however, only a total of 27 variants were observed in these genes (Fig. 1B). Most mutations were nonsense (fig. S3A), and the most prevalent nucleotide substitution was C>A (fig. S3B). The distribution of VAFs by mutated lymphoid driver genes and variant consequences is presented in fig. S3 (C and D). Most of the participants with lymphoid driver variants had only one variant (fig. S3E). The median VAFs were lower for putative lymphoid driver versus myeloid CHIP variants (6.8% versus 9.1%, *P* < 0.001; fig. S3F), consistent with lymphoid driver variants likely occurring in lymphoid progenitor/differentiated cells versus CHIP variants occurring at stem/progenitor cell level.

We also called mCAs in 8414 FINRISK participants with available SNP array genotyping data (Materials and Methods) and found 406 autosomal mCAs in 343 participants (table S5 and fig. S4). We identified loss of sex chromosomes as the most common mCAs and copy neutral loss of heterozygosity (CN-LOH) in chromosomes 1, 2, 9, and 14 as the most prevalent autosomal mCAs (fig. S4). CHIP significantly co-occurred with lymphoid drivers [odds ratio (OR) = 1.69, *P* = 0.03] as well as with autosomal mCAs (OR, 2.02; *P* = 0.001) in multivariable models. Lymphoid drivers did not significantly co-occur with autosomal mCAs (OR, 1.36; *P* = 0.26) (Fig. 1C).

### CH and clinical parameters in FINRISK

The prevalence of CHIP was strongly age related, whereas the prevalence of lymphoid drivers showed no evident age association (Fig. 1D). CHIP prevalence reached 7.8% [95% confidence interval (CI), 6.6 to 9.1%] in participants aged 60 to 69, consistent with the previously reported prevalence in population cohorts (*31*).

We systematically evaluated the association between CH and laboratory variables measured as part of FINRISK (fig. S5A). Participants with CHIP or autosomal mCAs had higher C-reactive protein (CRP) levels in univariable models (fig. S5C), and the association between CHIP and CRP remained statistically significant in a multivariable model (Fig. 1E). These results are in line with previous findings (*37*), suggesting that CHIP may be linked to inflammatory processes.

As expected, myeloid and lymphoid driver variants were associated with prevalent and incident hematologic malignancies (fig. S6, A and B). Furthermore, CHIP mutations were positively associated with heavy (more than 20 pack-years) smoking history that was mostly driven by mutations in *ASXL1*, as previously reported (*38*, *39*). Autosomal mCAs were also associated with heavy smoking history (fig. S5B).

### Enrichment of CHIP in FINRISK participants with RA

After excluding participants with prevalent hematologic malignancies, 1.2% (121 of 10,021) FINRISK participants had prevalent RA at DNA sampling. The presence of CHIP mutations was significantly associated with prevalent RA (OR, 2.06; *P* = 0.029; 95% CI, 1.08 to 3.94) in a multivariable model (Fig. 1F). This association was specific to participants with maximum VAF of CHIP variants less than 10% (OR, 3.00; *P* = 0.004; 95% CI, 1.41 to 6.35) and was driven by non-*DNMT3A* CHIP mutations (Fig. 1F). The lack of association between CHIP variants higher than 10% and RA was likely due to the low number of participants in this subset; only three participants with larger (more than 10% VAF) CHIP had prevalent RA in the cohort. The association was validated in questionnaire-based annotation of prevalent RA (fig. S7A). In contrast, the presence of lymphoid driver variants or mCA subtypes, including myeloid and lymphoid mCAs (*17*) were not associated with prevalent RA (Fig. 1F and fig. S7). Nine patients with CHIP developed incident RA during follow-up [Hazard ratio (HR), 1.62; 95% CI, 0.82 to 3.21; *P* = 0.16] (fig. S7B). We observed no difference in overall survival (OS) by CHIP status independent of RA (HR, 1.20; *P* = 0.08; fig. S6C) or in those with prevalent RA (HR, 0.55; *P* = 0.44). Together, CHIP is more common in participants with prevalent RA in the FINRISK cohort.

### Deriving CHIP status in SNP arrays

**Fig. 2.**
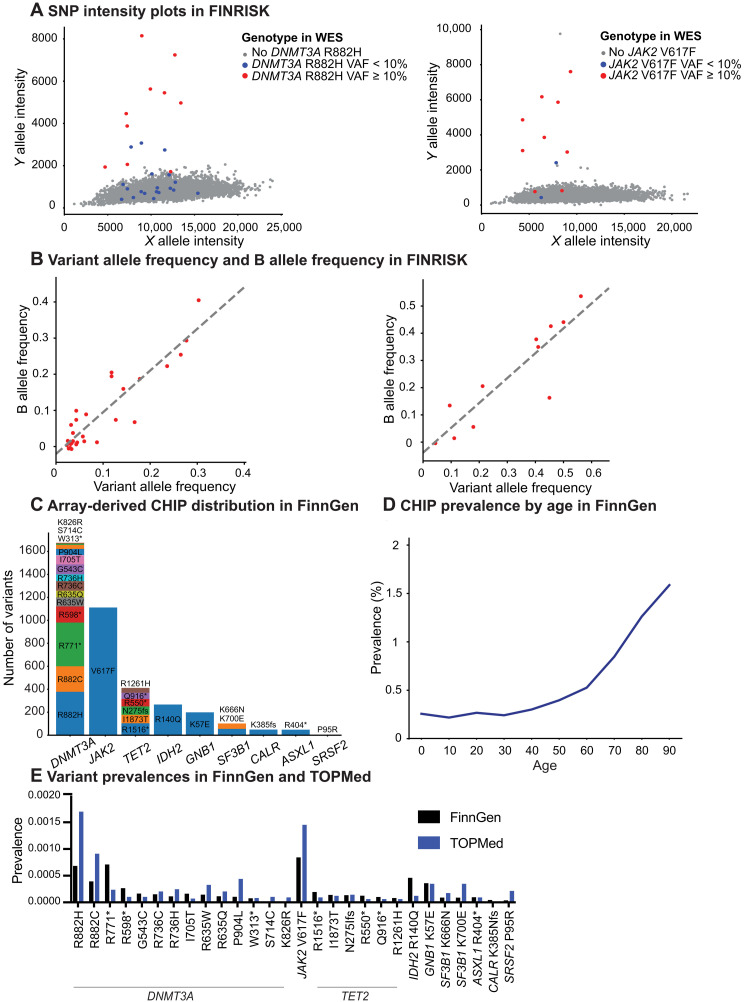
Detection of array-derived CHIP in FinnGen. (**A**) SNP intensity plots of CHIP hotspot variants *DNMT3A* R882H and *JAK2* V617F in FINRISK. Blue dots represent participants with the variant detected in WES with VAF < 10%, red dots represent VAF ≥ 10%, and gray dots represent no detected variant in WES. (**B**) Correlation of SNP array–derived B allele frequencies and WES-derived VAFs at *DNMT3A* R882H and *JAK2* V617F in FINRISK. *P* values calculated using Wald test. (**C**) Distribution of array-derived CHIP hotspot variants in FinnGen. Loci are ordered on the basis of the number of variant carriers in each amino acid. (**D**) Array-derived CHIP prevalence by age in FinnGen, excluding prevalent hematologic malignancies. (**E**) Comparison of detected hotspot variant prevalence between FinnGen and TOPMed, excluding prevalent hematologic malignancies.

DNA SNP arrays have been used to derive CHIP carriers based on outlier status in intensity plots at loci covered by the array (*8*). To systematically evaluate the concordance between array-derived CHIP status and CHIP detection with NGS, we analyzed *JAK2* V617F and *DNMT3A* R882H, the two most recurrent CHIP hotspot loci (*31*), in SNP intensity and WES data in the FINRISK cohort. Eight of the 11 (74%) CHIP carriers in *JAK2* V617F and 12 of the 29 (41%) CHIP carriers in *DNMT3A* R882H based on WES were also independently captured by detecting outliers in the SNP intensity plots (Fig. 2A and table S6). Variants at *JAK2* V617F and *DNMT3A* R882H with a VAF of 10% or more were detected with 77 and 90% likelihood in the SNP array data, respectively (Fig. 2A and table S6). Among participants with CHIP in FINRISK WES data, SNP array–derived relative B allele frequencies showed a strong correlation with WES-derived VAFs in both CHIP hotspots analyzed (Fig. 2B). On the basis of these analyses in the FINRISK cohort, we estimate that a high proportion (80 to 90% with VAF > 10%) of CHIP hotspot variants covered in the SNP array can be detected by manual curation of SNP intensity plots in a clone size-dependent manner.

The FinnGen cohort consists of SNP array data and registry-level clinical information for more than 500,000 Finnish participants, covering almost 10% of the Finnish population (*29*). The relatively old participant age (median, 55 years) and the high proportion of hospital-based recruitment facilitate enrichment of prevalent disease endpoints in FinnGen (*29*). A total of 41 CHIP hotspot loci with at least 10 variants in the UKBB (*31*) were covered by the FinnGen SNP array (table S7). We manually inspected intensity plots in all these sites and used heuristics, including variant prevalence relative to UKBB data (*31*) and associations with age and hematologic malignancies, to identify 28 high-confidence CHIP loci (Materials and Methods, table S7, Fig. 2C, and fig. S8). As expected, *JAK2* V617F had the highest CHIP hotspot prevalence, followed by *DNMT3A* R882H/C and R771* (Fig. 2C). The prevalence of FinnGen participants with array-derived CHIP reached 2.0% by age of 90 years (Fig. 2D and fig. S9A). CHIP carrier status was associated with prevalent and incident hematologic malignancies (fig. S9, B and C), but not with higher CRP levels (fig. S9, E to G). Nine hundred fifty-one of a total of 3882 (24.5%) variants, including 679 of the 1112 (61%) variants in the *JAK2* V617F hotspot, were found in participants with prevalent hematologic malignancies (fig. S9D). As an additional control, we confirmed similar prevalence of variants in our high-confidence loci with previously published whole-genome sequencing (WGS) data that robustly detected CHIP variants with >10% VAF from the Trans-Omics for Precision Medicine (TOPMed) cohort (Fig. 2E) (*32*). The median age of participants was 55 years in both FinnGen and TOPMed cohorts. In TOPMed (*32*), 758 of all 4180 CHIP variants (18.1%) were in the hotspot loci covered by the FinnGen array that passed our filtering criteria. Therefore, we estimate that 15 to 20% of all CHIP variants detected in WGS can also be detected in the FinnGen SNP array. Together, detecting CHIP hotspot variants in FinnGen SNP array data is feasible but is unable to capture non-hotspot variants and has limited sensitivity for smaller clones and for certain hotspot variants.

### Increased prevalence of CHIP in FinnGen participants with RA

**Fig. 3.**
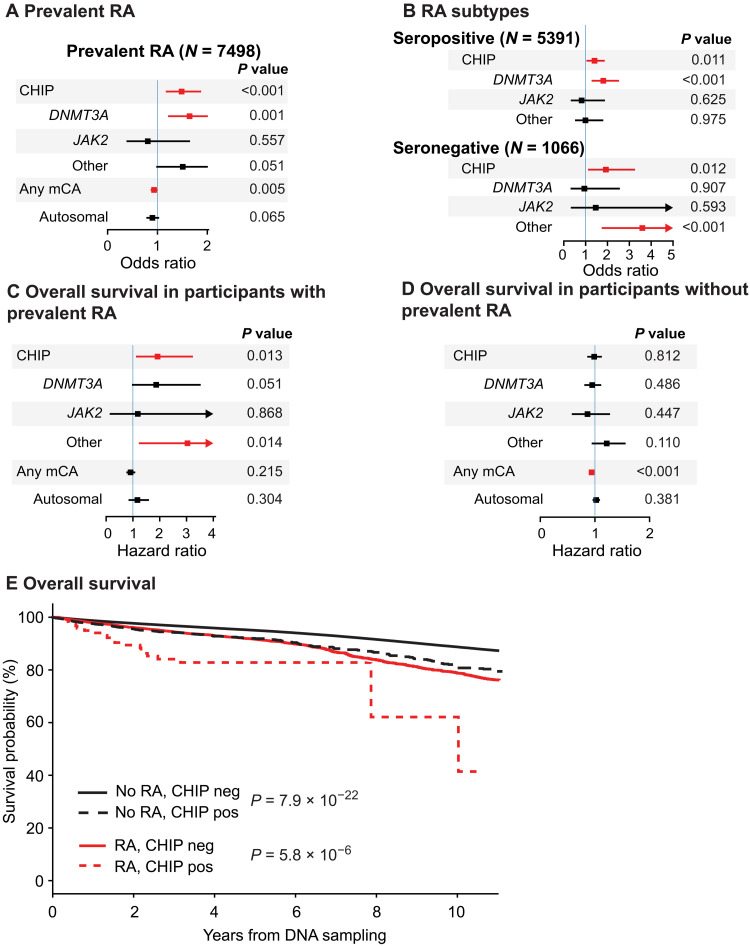
Association between CHIP and RA in FinnGen. (**A**) Association between CHIP and RA diagnosis before sampling. (**B**) Association between CHIP and RA subtype diagnosis before sampling. (**C**) Cox proportional hazards (Cox-PH) model for OS in FinnGen participants with prevalent RA diagnosis. (**D**) Cox-PH model for OS in FinnGen participants without prevalent RA diagnosis. [(A) to (D)] Adjusted for age, sex, smoking, and principal components for ancestry and excluded or censored for hematologic malignancies. In addition, the multivariable model in (C) was adjusted for time from RA diagnosis to sampling. (**E**) Kaplan-Meier for OS in FinnGen participants. [(C) to (E)] Outcomes calculated from time of DNA sample collection.

A total of 12,636 (2.5%) FinnGen participants had a history of RA diagnosis before DNA sampling (fig. S10A). Consistent with results in the FINRISK cohort, CHIP carrier status was associated with prevalent RA in a multivariable model in FinnGen (OR, 1.49; *P* < 0.001; 95% CI, 1.19 to 1.85) (Fig. 3A and fig. S10, B to D). We observed no positive association between prevalent RA and autosomal mCAs, including myeloid or lymphoid mCAs (Fig 3A and fig. S11). With 3655 incident RA cases, we found no difference in the cumulative incidence of RA by CH status in the FinnGen cohort (fig. S12).

Array-derived CHIP status was associated with both prevalent seropositive (*N* of prevalent seropositive cases, 5391; OR, 1.41; 95% CI; 1.08 to 1.83; *P* = 0.011) and seronegative (*N* of prevalent seronegative cases, 1066; OR, 1.93; 95% CI, 1.15 to 3.22; *P* = 0.012) RA (Fig. 3B). CHIP carrier status in *DNMT3A* was specifically associated with seropositive RA (OR, 1.81; 95% CI, 1.31 to 2.49; *P* < 0.001), whereas non-*DNMT3A*, non-*JAK2* CHIP was associated with seronegative RA (OR, 3.60; 95% CI, 1.78 to 7.27; *P* < 0.001) in multivariable models (Fig. 3B).

### Shorter survival in FinnGen participants with CHIPand prevalent RA

Among FinnGen participants with prevalent RA, CHIP variants conferred inferior OS when evaluated from the time of DNA sampling in a multivariable model (HR, 1.92; 95% CI, 1.15 to 3.20; *P* = 0.011; Fig. 3C). Array-derived CHIP was not associated with OS in FinnGen participants without RA (HR, 0.99; 95% CI, 0.88 to 1.11; *P* = 0.812; Fig. 3D), suggesting that CHIP may decrease survival specifically in patients with RA. The 5-year OS from DNA sampling for patients with RA was 83% in CHIP carriers versus 92% in those without CHIP (Fig. 3E). A total of 15 participants with prevalent RA and CHIP died during follow-up; eight (53%) were due to cardiovascular cause [International Classification of Diseases (ICD) codes I00-I99]. However, the CVD-specific mortality was not statistically significantly increased in CHIP carriers among participants with prevalent RA (HR, 1.77; *P* = 0.11).

### CHIP in newly diagnosed RA

To further evaluate the clinical impact of CHIP on RA phenotypes and outcomes, we collected DNA samples from four distinct RA patient cohorts of newly diagnosed, previously untreated, patients with RA without hematologic malignancies (table S8). A total of 573 patients with RA and 163 healthy controls underwent targeted NGS using a sequencing panel consisting of 65 genes recurrently mutated in myeloid malignancies (table S9). We also included a previously published cohort of 59 patients with RA with CHIP information in our analyses (*27*). The median age of patients with RA was 63 years.

**Fig. 4.**
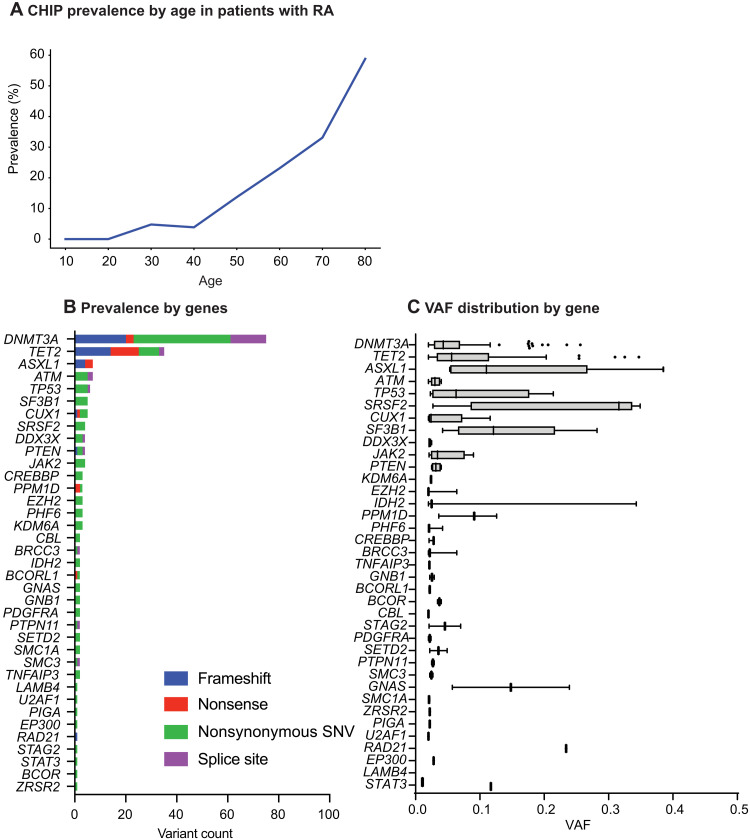
Spectrum of CHIP mutations in newly diagnosed RA. (**A**) CHIP prevalence by age in the RA patient cohort (*n* = 632). (**B**) Distribution of CHIP variants by gene and variant type. (**C**) Tukey box plot of VAFs by mutated gene in the RA patient cohort. Cohort characteristics are listed in Table 1 and in table S8.

We identified total of 204 CHIP variants in 134 patients with RA; 99 of those patients only had one variant (fig. S13, A and B, and table S10). The overall prevalence of CHIP variants in patients with newly diagnosed RA was 22% (126 of 573; Fig. 4A and fig. S13D). As expected (*5*), the most commonly mutated genes were *DNMT3A*, *TET2*, and *ASXL1* (Fig. 4B). We found the highest median VAFs in *ASXL1*, *SRSF2*, *SF3B1*, and *GNAS* (Fig. 4C). We observed no differences in CHIP prevalence between patients with RA and healthy controls (fig. S13C).

Next, we evaluated RA patient characteristics by CHIP status. The median age of patients with RA with CHIP was 69 years versus 62 years in patients with RA without CHIP (*P* < 0.001) (Table 1). While we observed no differences in CRP or erythrocyte sedimentation rate (ESR) (Table 1), the level of functional ability was lower, as indicated by higher health assessment questionnaire (HAQ) scores in patients with RA with CHIP versus those with no CHIP (*P* = 0.01) (Table 1). The association with higher HAQ scores remained significant when adjusting for age and sex (*P* = 0.009). The prevalence of comorbidities was similar between CHIP carriers and patients without CHIP (Table 1). Among 15 participants with coronary artery computed tomography scan available as part of the Early RA CVD study, we observed no difference by CHIP status in the coronary artery score (*P* = 0.44) (fig. S14).

**Table 1.** Patient characteristics of patients with newly diagnosed RA. Characteristics of patients with newly diagnosed RA by CHIP status in RA patient cohort (table S8). BMI, body mass index (kilograms per square meter); RF, rheumatoid factor (yes/no); ACPA, anti-citrullinated peptide antibody (yes/no); CRP, C-reactive protein (milligrams per liter); ESR, erythrocyte sedimentation rate (millimeters per hour); DAS, disease activity score (DAS28 score); HAQ, health assessment questionnaire disability index; AUC, area under the curve; TIA, transient ischemic attack.

	*n* patients withinformation available	No CHIP (*n* = 447)	CHIP (*n* = 126)	*P* value
Patient characteristics				
Age at diagnosis, median (range)	573	62 (18–86)	69 (38–89)	5.74E-10
Male sex, *n* (%)	573	162 (36)	47 (37)	0.83
Smoking history, *n* (%)	487	221 (60)	60 (51)	0.11
BMI, median (range)	489	26.1 (17.2–44.8)	26.1 (17.0–37.2)	0.98
RA characteristics				
RF+, *n* (%)	493	235 (63)	69 (58)	0.39
ACPA+, *n* (%)	495	229 (61)	65 (55)	0.24
Seropositive, *n* (%)	555	302 (70)	80 (64)	0.19
Baseline CRP, median (range)	545	11 (0–191)	14 (0–147)	0.81
Baseline ESR, median (range)	555	26 (1–110)	28 (2–109)	0.16
Baseline DAS28, median (range)	573	4.91 (0.77–8.23)	5.28 (1.68–7.83)	0.07
HAQ, median (range)	507	0.88 (0–2.75)	1.13 (0–2.63)	0.01
DAS28 24-month AUC, median (range)	450	83 (39–147)	86 (33–146)	0.94
Comorbidities				
Diabetes, *n* (%)	103	2 (4)	4 (14)	0.19
Hypertension, *n* (%)	489	143 (38)	48 (44)	0.27
Chronic heart disease, *n* (%)	103	0 (0)	1 (7)	0.13
TIA/stroke, *n* (%)	103	1 (1)	1 (7)	0.25
Coronary artery disease, *n* (%)	103	4 (4)	1 (7)	0.53

**Fig. 5.**
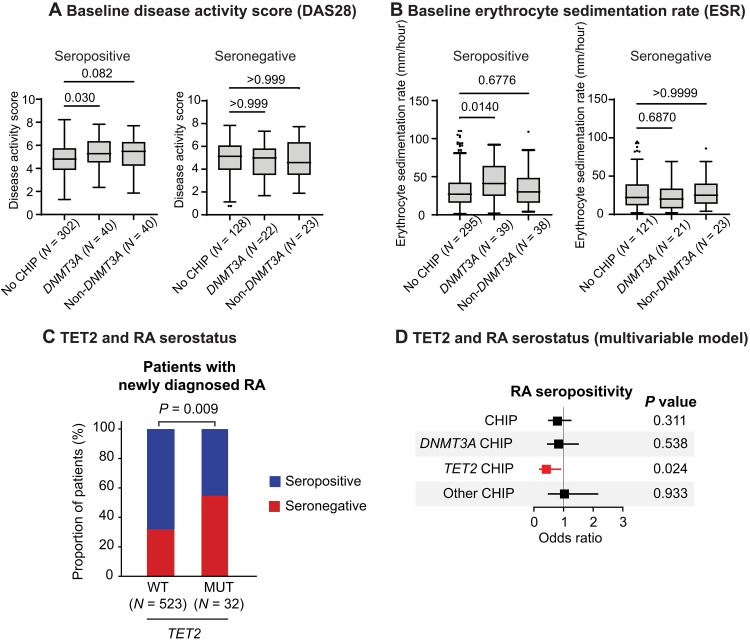
RA phenotypes at diagnosis associated with CHIP. (**A**) Disease activity scores by CHIP status in seropositive and seronegative RA. (**B**) ESRs by CHIP status and RA serotype. [(A) and (B)] *P* values calculated using Kruskal-Wallis test. (**C**) Proportion of patients with seronegative RA by *TET2* CHIP status. The *P* value was calculated using Fisher’s exact test. (**D**) Multivariable model for CHIP subtypes and RA seropositivity adjusted for age, sex, and smoking.

While the presence of any CHIP was not significantly associated with RA disease activity, we hypothesized that specific gene mutations may be associated with inflammation and enriched in patients with seropositive and seronegative disease subtypes. Patients with *DNMT3A* mutations had significantly higher ESR and disease-activity scores specifically in patients with seropositive, but not with seronegative, RA (Fig. 5, A and B), suggesting that *DNMT3A* may contribute to increased inflammatory activity in patients with seropositive RA. The associations between *DNMT3A* and higher ESR and DAS28 were independent of age and sex (fig. S15, A and B). In contrast, *TET2* mutations were significantly more common in patients with seronegative RA both in a univariable (Fig. 5C and fig. S15C) and in a multivariable model that adjusted for age, sex, and smoking (Fig. 5D). A similar trend was observed across RA subcohorts and when adjusting for ESR and CRP (fig. S15, D to F). We observed no differences in seronegative patient characteristics by *TET2* CHIP status (table S11).

### CHIP and outcomes in RA

**Fig. 6.**
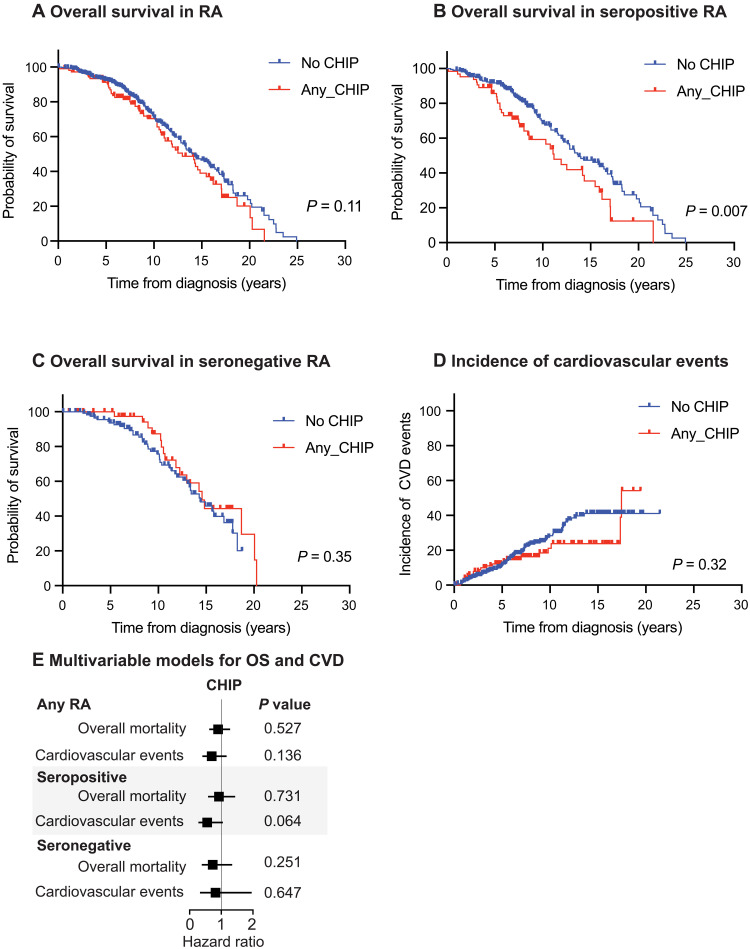
RA patient outcomes by CHIP status. (**A**) Kaplan-Meier curve for OS in patients with RA with and without CHIP. (**B**) Kaplan-Meier curve for OS in patients with seropositive RA with and without CHIP. (**C**) Kaplan-Meier curve for OS in patients with seronegative RA with and without CHIP. (**D**) Cumulative incidence of cardiovascular events in patients with RA. [(A) to (D)] *P* values calculated using Mantel-Cox test. (**E**) Cox-PH models for OS and cardiovascular events in patients with RA. Adjusted for age, sex, and smoking.

The area under the curve for DAS28 during 24 months after RA diagnosis was similar between patients with RA with and without CHIP (Table 1). Among 448 patients with RA with long-term clinical follow-up, the median OS was 14 years. Patients with CHIP had similar OS compared with patients without CHIP (Fig. 6A). However, we observed worse OS in patients with seropositive RA with CHIP compared to seropositive patients without CHIP (Fig. 6B). No difference in OS by CHIP status was observed in seronegative patients (Fig. 6C). Furthermore, the cumulative incidence of cardiovascular events was similar in patients with and without CHIP (Fig. 6D). No differences in OS or incidence of cardiovascular events by CHIP status were seen in multivariable models when adjusting for age, sex, and smoking history (Fig. 6E).

## DISCUSSION

Our study adds to a growing body of evidence linking chronic inflammation with clonal HSC expansion in a context-specific manner (*6*). In our data, CHIP was associated with prevalent RA in two population-level cohorts, consistent with smaller prior studies (*25*, *26*). Understanding the interplay between age-related inflammatory diseases and CH may result in personalized therapeutic opportunities, as in the case of CANTOS trial, where carriers of *TET2* mutations were more likely to benefit from interleukin-1β (IL-1β) inhibition for secondary CVD prevention (*14*). IL-1β inhibition also belongs to the armamentarium of approved targeted therapies for RA (*20*), and IL-1β blockage can suppress the expansion of *Tet2*-mutated HSCs in mice (*12*). While we observed no differences in OS, CVD risk, or clinical remission rates by CHIP status in patients with RA in multivariable models, the relative efficacy of IL-1β inhibition in RA subtypes by CHIP status could be tested in future clinical trials for personalized therapy.

A previous study in the UKBB identified a nominal association between CHIP and incident RA that did not reach phenome-wide significance (*31*). In addition, a recent preprint article leveraged WES and WGS data of more than 650,000 participants in three large-scale biobank cohorts and showed that CHIP clones increase the risk of incident RA (*40*). In our study, CHIP was not associated with incident RA, potentially due to the limited cohort size in the FINRISK cohort and to the relatively short follow-up time in the FinnGen study (median follow-up since DNA sampling, 4 years).

Our analyses in the FinnGen study and in the RA patient cohort identified previously unknown genotype-phenotype associations between CHIP and RA. *DNMT3A*-mutated CHIP was more common in prevalent seropositive RA in the FinnGen study, and patients with newly diagnosed seropositive RA with *DNMT3A* mutations had higher DAS28 disease severity score as well as higher ESR. Consistent with these results, a recent study by Wang and colleagues reported more severe arthritis phenotype and higher infiltration of *Dnmt3a*-mutated immune cells in the synovia of mice transplanted with HSCs carrying the *Dnmt3a* hotspot mutation and exposed to a collagen antibody-induced arthritis model (*41*). In contrast, non-*DNMT3A* variants were significantly more common in FinnGen participants with prevalent seronegative RA. In patients with newly diagnosed RA, *TET2*-mutated CHIP was significantly enriched in the seronegative disease subtype. While seronegative RA is a heterogeneous disease entity (*20*), the disease subtypes were carefully annotated by the treating rheumatologist in our RA patient cohorts. Production of IL-1β from proinflammatory monocytes may act as a key mediator of inflammation in RA (*42*), and innate immunity is thought to play a more significant role in the pathogenesis of seronegative RA (*43*, *44*). *Tet2*-deficient macrophages have shown increased production of proinflammatory cytokines, particularly IL-1β, in murine models of gout (*7*) and CVD (*11*, *45*), and *TET2* mutations were associated with higher IL-1β levels in the TOPMed study (*32*). Therefore, we postulate that CHIP, especially driven by *TET2* mutations, may contribute to disease pathophysiology of seronegative RA by increasing innate immune cell activation. Intriguingly, a recent study identified proinflammatory M1 macrophages with up-regulated *IL1B* gene expression in the synovia of patients with ACPA-negative versus ACPA-positive RA (*46*); the potential contribution of CHIP clones to this macrophage phenotype is an exciting area of future research. On the other hand, systematic inflammation in seronegative RA may selectively promote expansion of CHIP clones in the bone marrow and underlie the association between seronegative RA and CHIP. However, care must be taken when inferring causality from associations between two aging-related phenomena, such as RA and CHIP, as these may be explained by confounding factors such as “biological age” and not by causal relationships. Subsequent studies with longitudinal blood sampling pre– and post–RA diagnosis may help evaluate temporal clonal dynamics during disease development and upon anti-inflammatory therapy as well as the functional role of CHIP in various RA subtypes and its potential therapeutic implications, including effects on long-term outcomes.

Notably, CHIP was associated with RA and its phenotypes across cohorts, despite the variability in CHIP detection methods used in our study, including WES, SNP arrays, and targeted NGS. The SNP array–based annotation of CHIP carriers in FinnGen only captures a minor subset of CHIP variants located in the recurrent hotspot loci and is unable to detect the full spectrum of variants in CHIP genes. Our results from the FINRISK cohort suggest that SNP arrays reliably detect CHIP variants with VAF greater than 10%. Furthermore, when comparing WGS data from the TOPMed study (*32*), we estimated that 18% of all CHIP variants detected in WGS can also be detected in the FinnGen SNP array. Therefore, when available, WES and WGS remain superior methods to capture the entire spectrum of CHIP in population-level cohorts.

Although the random population-level sampling of the FINRISK study and the hospital-based recruitment in FinnGen provided complementary cohorts to evaluate the association between CH and RA, the limited cohort size of FINRISK and the absence of NGS data in FinnGen highlight the need for further analyses of the link between CHIP and prevalent autoimmune disease in other populations and cohorts. Smaller CHIP clones (under 10% VAF) were specifically associated with prevalent RA in the FINRISK cohort; however, only three participants with prevalent RA had CHIP clones with more than 10% VAF. While the lack of association between larger CHIP clones and prevalent RA can be explained by the relatively small cohort size, we are unable to exclude the possibility of noncausal association behind these observations. Also, the FINRISK cohort was not powered to detected differences between specific CHIP genes and RA subtypes. On the other hand, our array-based CHIP analysis can only detect larger clones and mutations in hotspot loci in the FinnGen cohort, which may bias the spectrum of CHIP mutations observed in RA subtypes.

Together, our analyses using population-level and disease-specific cohorts point to a context-dependent association between CHIP and distinct RA subtypes. *DNMT3A* mutations were associated with a more severe clinical phenotype of seropositive RA, whereas *TET2*-mutated CHIP was enriched in the seronegative RA. Further studies are needed to delineate the clinical and functional relevance of CHIP detection in patients with RA.

## MATERIALS AND METHODS

### FINRISK cohort

The FINRISK study is a Finnish population-level cohort consisting of random population-level sampling of individuals aged 25 to 74 (*28*). We analyzed disease endpoints from Finnish national health registries, laboratory measurements, and questionnaire-based information for the FINRISK participants recruited between 1992 and 2007.

### Somatic variant calling of CHIP and lymphoid drivervariants in FINRISK

We obtained WES data for 10,129 previously sequenced FINRISK participants (*30*). We called CH variants from the WES data using Illumina DRAGEN v3.8 in tumor-only mode. We generated a panel of normals using 40 FINRISK participants under 40 years of age, and these samples were confirmed not to have any putative CHIP variants. The panel of normals was then included in the Illumina DRAGEN variant calling algorithm. DRAGEN commands are listed in table S12. We used ANNOVAR (*47*) for variant annotation. To detect CHIP variants, we used a candidate list consisting of 82 genes (table S2). To filter benign and likely germline variation, we used a maximum population allele frequency threshold of 0.1% in WES of any population in gnomAD (*48*) (version 3.1.2), excluding known CHIP hotspots *JAK2* V617F and *DNMT3A* R882. We also required variants to be labeled “PASS” by DRAGEN, have a somatic quality score of 3 or higher, have a GermlineQuality score of less than 10, and have at least 10 reads aligning to the variant loci, out of which at least 2 are variant reads. At least one read on both strands supporting both alleles was required. Furthermore, splicing variants occurring further than 2 base pairs (bp) from the intron-exon boundary were excluded, as well as frameshift variants seen in more than 0.05% of samples. For frameshift variants occurring within 5 bp of each other, only the variant with higher VAF was included for downstream analysis. No VAF thresholds were used to filter variants; however, only two variants that passed through filters had VAF of less than 2% (table S3), consistent with 2% VAF being the detection limit in WES data (*3*). Last, we manually curated detected variants using Interactive Genomics Viewer (IGV). For lymphoid driver variants, we generated a list of 320 candidate genes described in table S4, containing the genes and candidate loci originally described by Niroula *et al.* (*17*) as lymphoid CHIP (L-CHIP) appended with known acute lymphoblastic leukemia (ALL) driver where variants were considered as putative drivers in a recent study by Brady *et al.* (*36*). Variants identified in these genes were filtered as above. In addition, for lymphoid driver variants, we required the VAFs to be between 2 and 35% and, for missense lymphoid driver variants, we required the variant to be reported at least three times in COSMIC or at least once in the context of L-CHIP (*17*) or ALL (*36*).

### FinnGen cohort

FinnGen is a research project (*29*) that has compiled genotype and phenotype information from 520,210 Finnish individuals (FinnGen Data Freeze 12). FinnGen samples are collected from six regional hospital biobanks, a national level biobank, a private sector biobank, and the Blood Service biobank. Most of FinnGen samples have been genotyped using FinnGen’s custom Thermo Fisher Scientific Axiom array, containing 736,145 probes for 655,973 loci. In addition, a subset of samples was genotyped using legacy chips from old studies. To further enhance the genetic information available, further SNPs were imputed using the SISu v.4.0 imputation panel. The genetic information available is combined with data collected from Finnish national health registries, including diagnosis codes.

### CHIP calling in FinnGen

CHIP hotspot variants were called in FinnGen by manually examining SNP intensity plots in coding areas of CHIP-associated genes. We queried candidate CHIP loci by including all coordinates of the FinnGen SNP array where at least 10 CHIP variants were identified in the WES data of 200,453 UKBB participants (*31*). Loci were further evaluated by calculating associations with age, hematologic malignancy, and the relative prevalence to detected variants in the publicly available UKBB WES data (fig. S8) (*31*). For candidate SNP loci in CHIP genes, filtering criteria were defined as follows: less than 10-fold prevalence of variants relative to UKBB data, positive univariate (*P* < 0.1) association with age, and positive univariate association (*P* < 0.05) with a hematologic malignancy diagnosis code at any point in the registry data (tables S7 and S13). We also analyzed myeloid, lymphoid, and other hematologic malignancy diagnosis codes separately. If the relative prevalence compared to that in UKBB was less than twofold, then we required one of the associations with age or hematologic malignancy. The relative prevalence of the variants was also compared with CHIP in the TOPMed WGS data (*32*).

### mCA detection in FINRISK and FinnGen

mCAs were called in FINRISK and FinnGen using the MoChA pipeline (*15*) with SHAPEIT4 (*49*) haplotype phasing. Samples with a call rate of less than 0.97 or BAF autocorrelation of more than 0.03 were excluded.

### Endpoint definitions in FINRISK and FinnGen

Disease endpoints were defined by querying national health registries for ICD codes according to the table S13. RA endpoints in FINRISK and FinnGen cohorts were annotated as seronegative only if there were no codes for seropositive RA in the registries, i.e., participants with both codes found in the registry were defined as seropositive.

### RA patient cohorts

We selected two distinct RA patient cohorts from the Biobank at the Rheumatology Department in Umeå, Sweden. Cohort 1 consisted of 150 patients with RA that were selected in a case-control manner for CVD (*50*). Cohort 2 consisted of 300 patients with early RA, half of which had seronegative (ACPA and RF negative) and half seropositive (ACPA and/or RF positive) RA subtypes (*51*). In addition, we included samples from 18 and 60 patients with RA from Finnish FIN-RACo (*52*) and NEO-RACo (*53*) clinical trials, respectively, as well as from a prospective cohort of 45 patients with blood sample collection at RA diagnosis (FosfoRA). Furthermore, we included 59 samples with CHIP information available, from a previously published cohort study on CHIP and RA by Savola *et al.* (*27*). We also sequenced 163 healthy controls collected from the Finnish Red Cross Blood Service. Blood donation criteria excluded all patients with hematological diseases and rheumatoid diseases in symptomatic phase or treated with other than NSAID or hydroxychloroquine. All participants provided informed consent to participate in the study. The median age of the patients with RA was 64 years (18 to 89), and the median year of diagnosis was 2006 (1993 to 2021). Sixty-five percent of patients with RA were female, and 434 of the 632 patients with RA were annotated as seropositive. Characteristics of the individual cohorts are listed in table S8.

### Targeted NGS panel to detect CHIP

We identified CHIP variants in patients with RA and healthy controls from the Finnish Red Cross Blood Service Biobank using panel sequencing of 65 myeloid driver genes (table S9). Genomic DNA (40 to 50 ng) was processed according to Twist Library Preparation EF 2.0 with Enzymatic Fragmentation DOC-001239 REV 1.0 and Twist Target Enrichment Protocol DOC-001085 REV 2.0 manual (Twist Bioscience, San Francisco, CA, USA) with the following modifications. IDT xGEN unique dual index with unique molecular identifier adapters were used for ligation (Integrated DNA Technologies, Coralville, IA, USA). Library quantification and quality check was performed using LabChip GX Touch HT High Sensitivity assay (PerkinElmer, Waltham, MA, USA) and Qubit Broad Range DNA Assay (Thermo Fisher Scientific, Waltham, MA, USA). Libraries were pooled to 14- to 16-plex reactions. The exome enrichment was performed using Twist custom panel probes (244 kb). The captured library pools were quantified for sequencing using QuantStudio5 Collibri Library Quantification kit (Thermo Fisher Scientific, Waltham, MA, USA) and LabChip GX Touch HT High Sensitivity assay (PerkinElmer, Waltham, MA, USA).

Sequencing was performed with Illumina NovaSeq 6000 system (Illumina, San Diego, CA, USA) and v1.5 chemistry. The median target sequencing coverage was 1700× across samples.

We used Illumina DRAGEN 3.8 in tumor-only mode for variant calling and ANNOVAR for variant annotation. The DRAGEN commands are listed in table S12.

Only variants labeled PASS by DRAGEN were included for subsequent filtering. We excluded variants with gnomAD maximum population allele frequency of more than 0.1% or occurring in more than 1% of our samples. We also excluded variants with less than 100 total reads or with less than two variant allele reads. We excluded variants with a VAF of more than 40%. Furthermore, we required every variant to have at least one read in each direction supporting each allele. Last, we included only variants with a VAF of at least 2% as CHIP.

### Statistical analysis

In each subcohort, genotypes were assigned before evaluating the association with clinical variables, including outcomes. Analyses were performed using Python and GraphPad Prism. For FinnGen data, analyses were performed in the dedicated Sandbox environment. Python package statsmodels 0.14.2 was used for fitting logistic regression models, lifelines 0.29.0 was used for fitting Cox proportional hazards (Cox-PH) and Kaplan-Meier models, and statistical tests implemented in SciPy 1.11.4 and GraphPad Prism 10.3.0 were used. *P* values reported are two sided. *P* values of less than 0.05 were considered statistically significant throughout the study, unless otherwise mentioned. Multivariable models were adjusted for age, sex, questionnaire-based smoking history (never/ever), and 10 principal components of ancestry, and participants with missing values in any of these were excluded, unless otherwise mentioned. Hematologic malignancies were excluded in co-occurrence models and right censored in survival models.

### Ethics statement

This study was approved by the ethics committees of the local university hospitals, and the principles of the Helsinki Declaration were followed. This study was conducted under the ethical approval number 181/13/03/01/12 from the Ethics Committee of HUS (Helsinki, Finland). All patients had given their written informed consent before sample collection.

## Supplementary Materials

### The PDF file includes:

Figs. S1 to S15 Legends for tables S1 to S13 List of FinnGen authors and affiliations
