# Supplementary material for: Clonal hematopoiesis is associated with distinct rheumatoid arthritis phenotypes
Source: Sci Adv. 2025 Apr 30;11(18):eadt9846. doi: 10.1126/sciadv.adt9846 (PMC12042900; doi:10.1126/sciadv.adt9846)
Supplement: Supplementary file 1 — Figs. S1 to S15 Legends for tables S1 to S13 List of FinnGen authors and affiliations [file sciadv.adt9846_sm.pdf]

Science Advances
AAAS

Supplementary Materials for

Clonal hematopoiesis is associated with distinct rheumatoid arthritis phenotypes

Emil Hiitola *et al.*

Corresponding author: Mikko Myllymäki, mikko.myllymaki@helsinki.fi

Sci. Adv. **11**, eadt9846 (2025)
DOI: 10.1126/sciadv.adt9846

The PDF file includes:

Figs. S1 to S15
Legends for tables S1 to S13
List of FinnGen authors and affiliations

Other Supplementary Material for this manuscript includes the following:

Tables S1 to S13

Figure S1

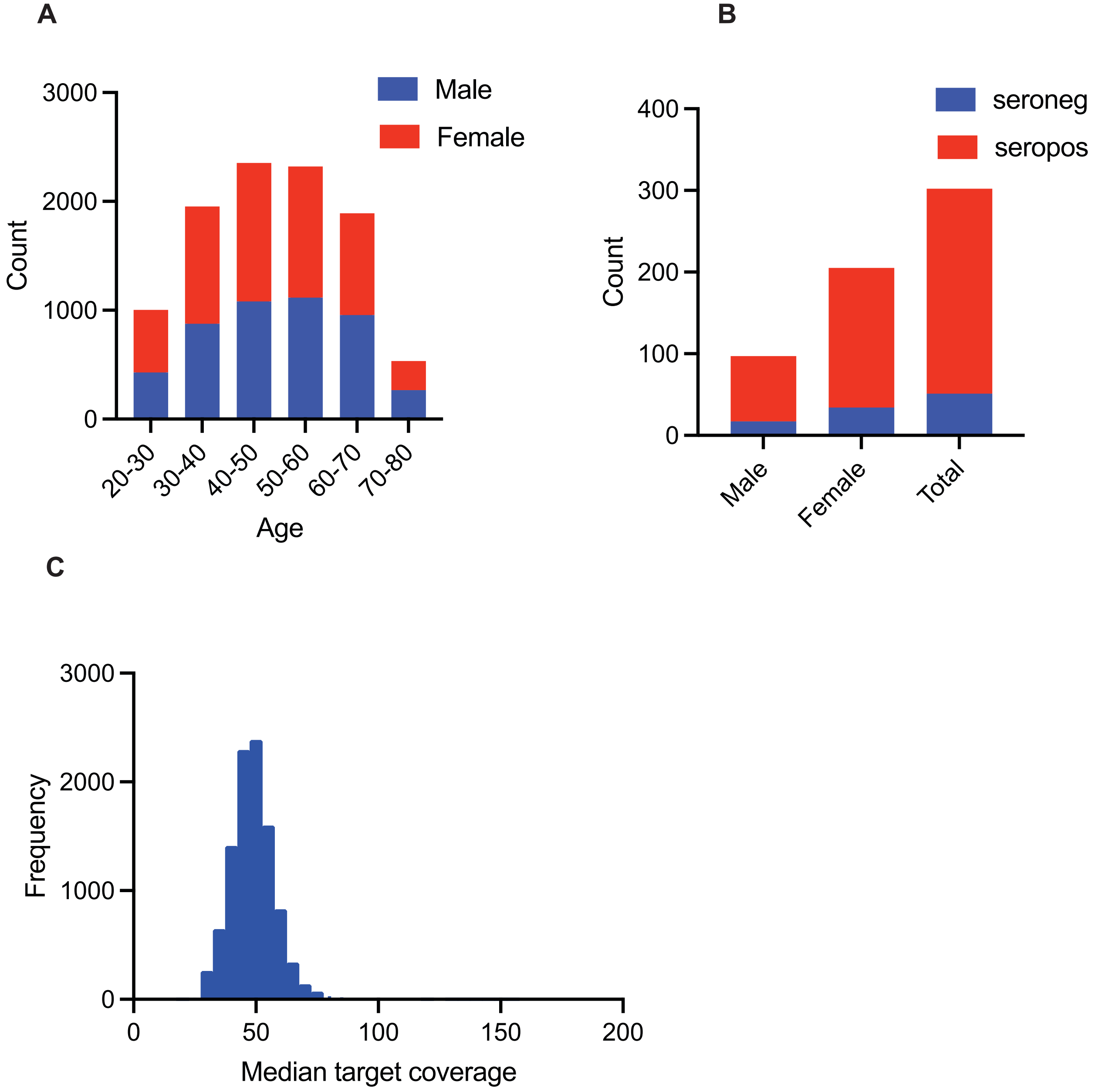

Figure S1: FINRISK cohort characteristics
(A) Age and sex distribution in the FINRISK cohort. (B) RA cases in FINRISK. (C) Distribution of median target coverages in FINRISK participants.

Figure S2

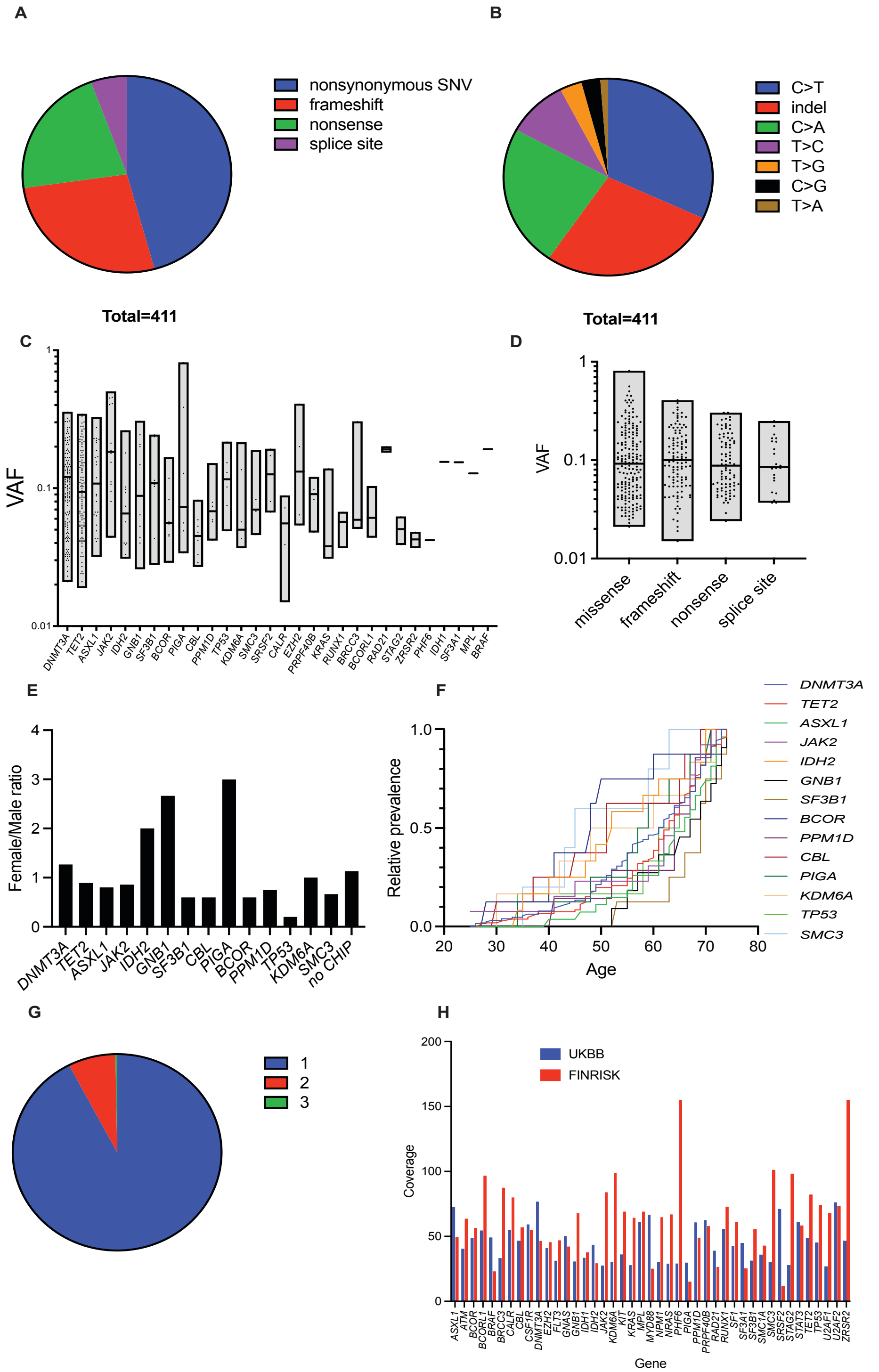

Figure S2: CHIP characteristics in FINRISK

(A) Distribution of CHIP variant consequences. (B) Distribution of nucleotide substitutions. (C) VAF distribution by gene. (D) VAF distribution by variant consequence. (E) Female/Male ratio by gene. (F) Empirical cumulative distribution of the most common gene variants by age. (G) Number of participants with 1, 2 or 3 CHIP mutations. (H) Comparison of coverages at CHIP gene exons in the FINRISK and the UKBB (from Kar et al. Nature Genetics 2022).

Figure S3

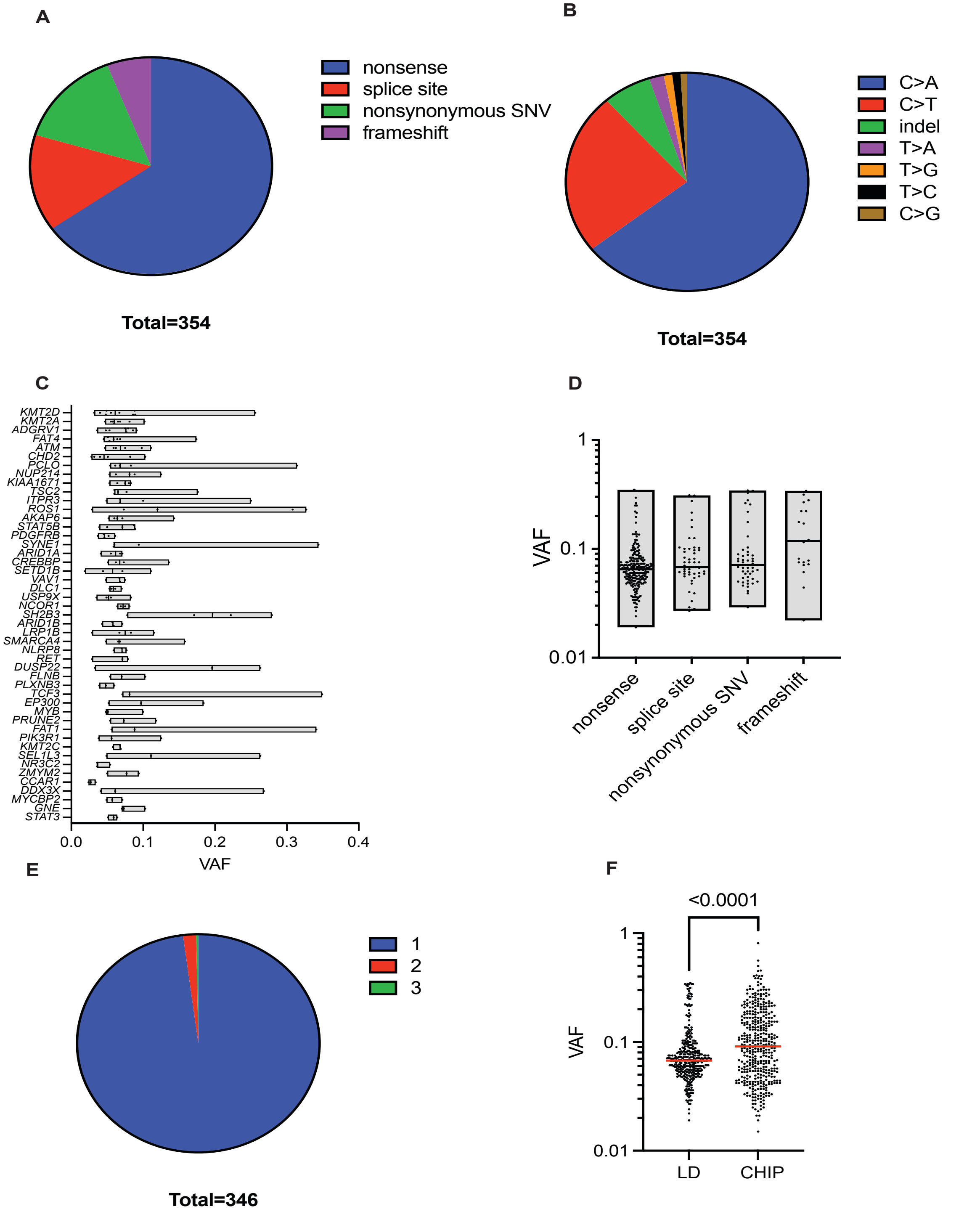

Figure S3: Lymphoid driver characteristics in FINRISK

(A) Distribution of lymphoid driver variant consequences. (B) Distribution of nucleotide substitutions. (C) VAF distributions by the most common lymphoid driver genes. (D) VAF distributions by variant consequence. (E) Number of participants with 1, 2 or more lymphoid driver variants. (F) CHIP vs lymphoid driver VAFs. C-F excluding prevalent hematologic malignancies.

Figure S4

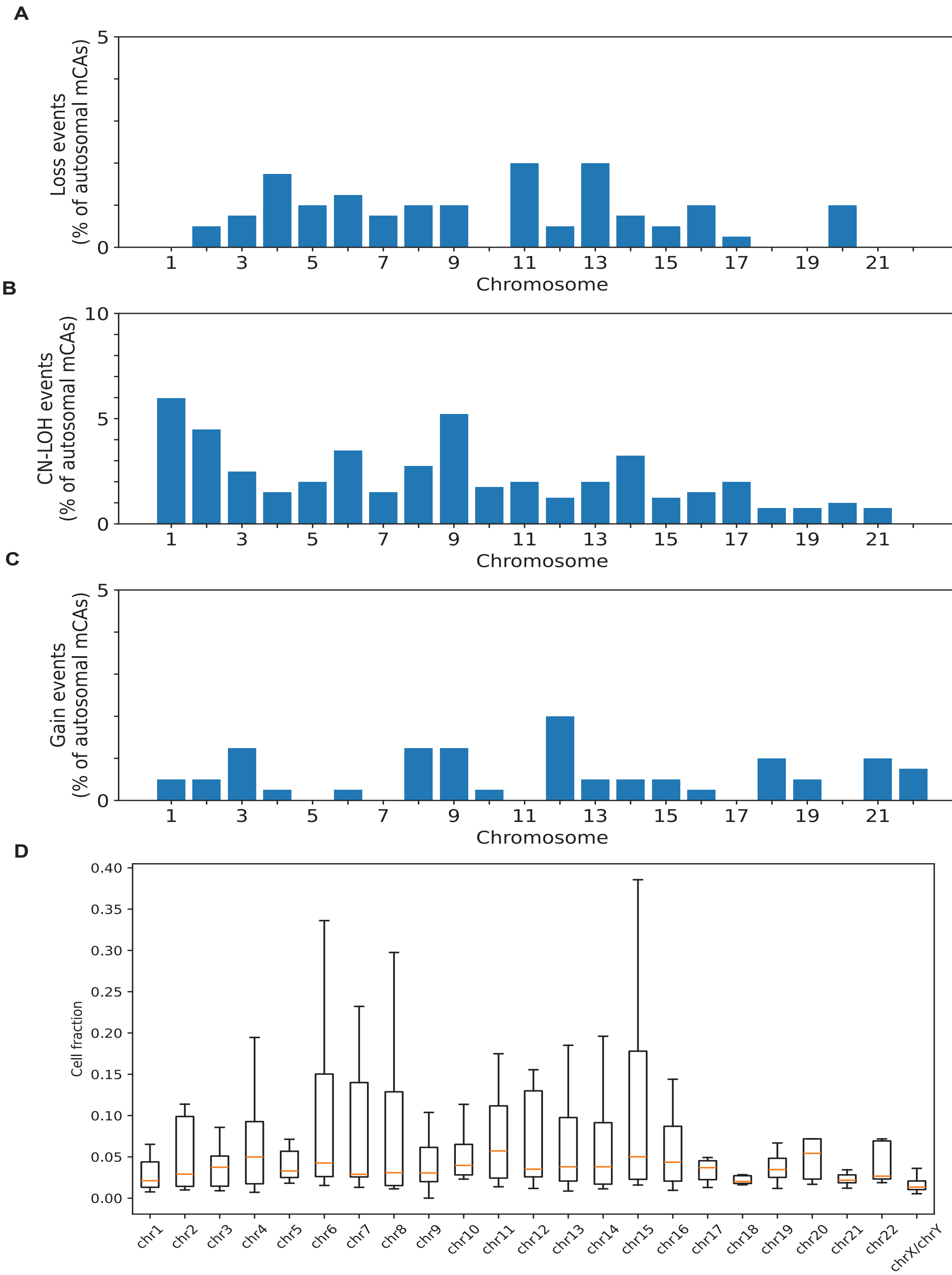

Figure S4: mCAs in FINRISK

(A) Distribution of autosomal loss events by chromosome. (B) Distribution of autosomal CN-LOH events by chromosome. (C) Distribution of autosomal gain events by chromosome. (D) Distribution of estimated cell fractions by chromosome.

Figure S5

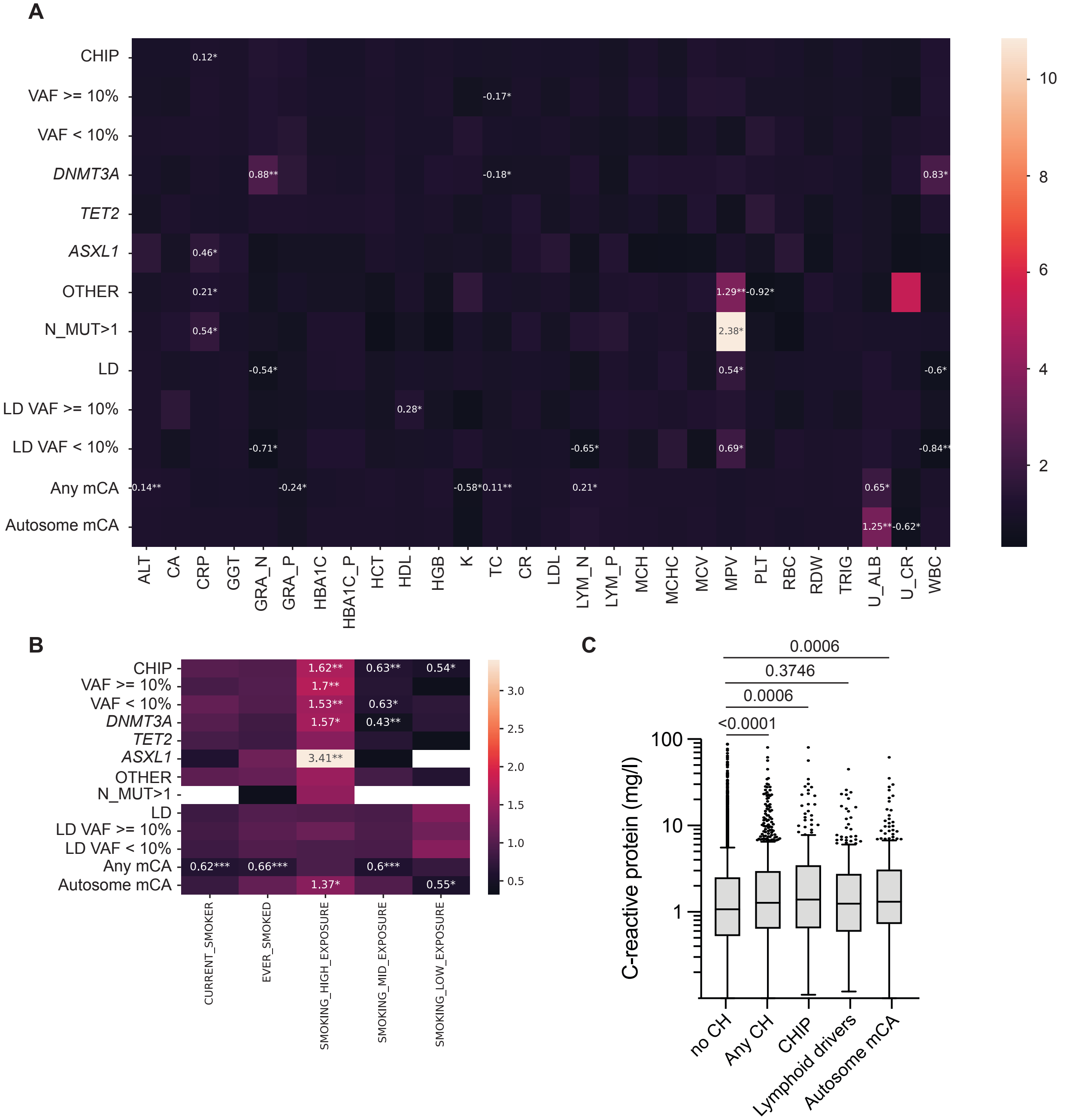

Figure S5: Associations between clonal hematopoiesis phenotypes and selected phenotypes in FINRISK

(A) Associations of between CH and normalized blood counts and biochemical measurements. Calculations performed using a generalized linear model adjusting for age, sex, smoking history and principal components of ancestry, and excluding hematologic malignancies. Effect sizes (beta) are indicated by the color scale and written out if the association is statistically significant. Beta reflects the difference in mean values between groups as number of standard deviations. (B) Associations between CH and smoking phenotypes. High, medium and low exposure defined as >=20, 5-20 and 0-5 pack-years. Fitted using a logistic regression model excluding prevalent hematologic malignancies, not adjusting for covariates. (C) Univariate association between CH

subtypes and C-reactive protein levels. P-values calculated using Mann-Whitney *U* test. A: ALT: alanine transaminase, CA: Serum calcium, CRP: C-reactive protein, GCT: Serum gamma-glutamyl transferase, GRA_N: Granulocytes (N), GRA_P: Granulocytes (%), HBA1C: Glycosylated hemoglobin, HBA1C_P: Glycosylated hemoglobin (%), HCT: Hematocrit level, HDL: Serum high-density lipoprotein, HGB: Hemoglobin, K: Urine potassium, TC: Serum cholesterol, CR: Serum creatinine, LDL: Serum low-density lipoprotein, LYM_N: Lymphocyte count, LYM_P: Lymphocytes (%), MCH: Mean cell hemoglobin, MCHC: Mean corpuscular hemoglobin, MCV: Mean erythrocyte volume, MPV: Platelet mean volume, PLT: Platelet count, RBC: Red blood cell count, RBV: Red blood cell size distribution width, TRIG: Serum triglyceride levels, U_ALB: Urine albumine, U_CR: Urine creatinine, WBC: White blood cell count. A-B: *P<0.05, **P<0.01, ***P<0.00001.

Figure S6

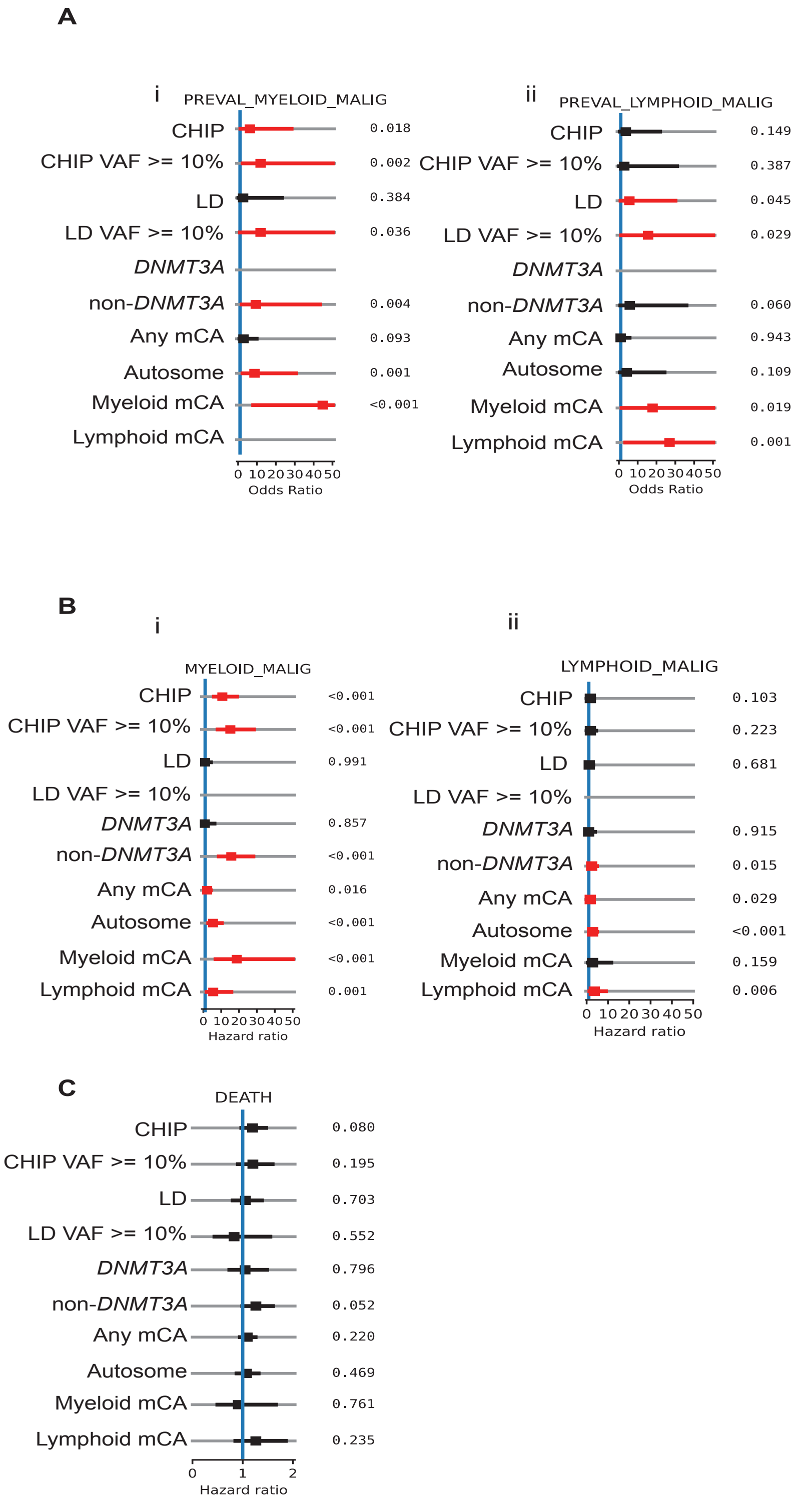

Figure S6: Associations between array-derived CHIP and outcomes
(A) Association between CH and prevalent (i) myeloid and (ii) lymphoid malignancy diagnosis, fitted using a logistic regression model adjusting for age, sex, smoking and 10 principal components. (B) Association between CH and incident (i) myeloid and (ii) lymphoid malignancy diagnosis, using Cox-PH models. (C) CH and overall survival.

Figure S7

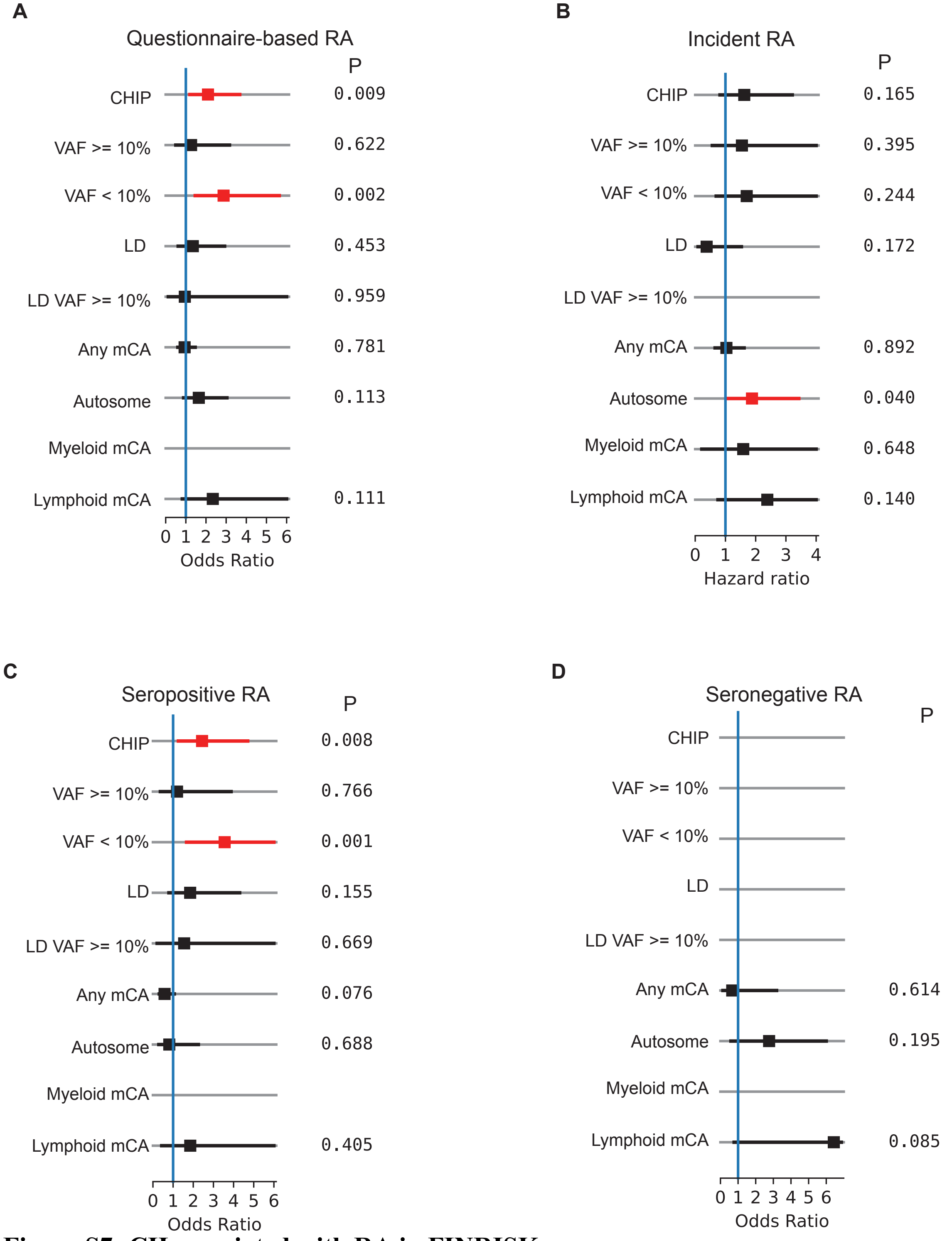

Figure S7: CH associated with RA in FINRISK

(A) CH associated with questionnaire-based RA. (B) CH associated with incident RA (Cox-PH model). (C) CH associated with seropositive RA. (D) CH associated with seronegative RA. Models adjusted for age, sex, smoking, 10 principal components and excluding/right censoring hematologic malignancies. Empty lines represent analyses with no events.

Figure S8

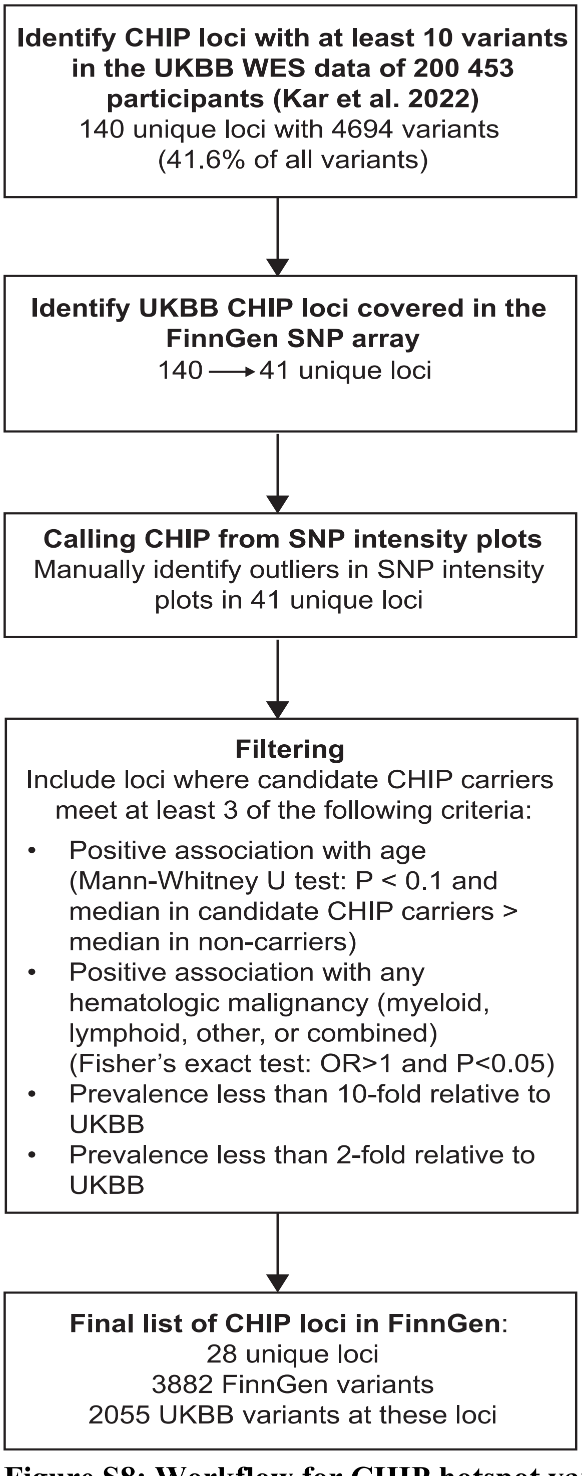

Figure S8: Workflow for CHIP hotspot variant calling in FinnGen

For any given CHIP locus, if the relative prevalence of CHIP variants compared to the UKBB was less than 2-fold, we required the variant carriers to be significantly associated with age or have a significant association with hematologic malignancies (or both). For CHIP loci with the relative prevalence between 2- to 10-fold, we required the variant carriers to be significantly associated with both age and hematologic malignancies.

Figure S9

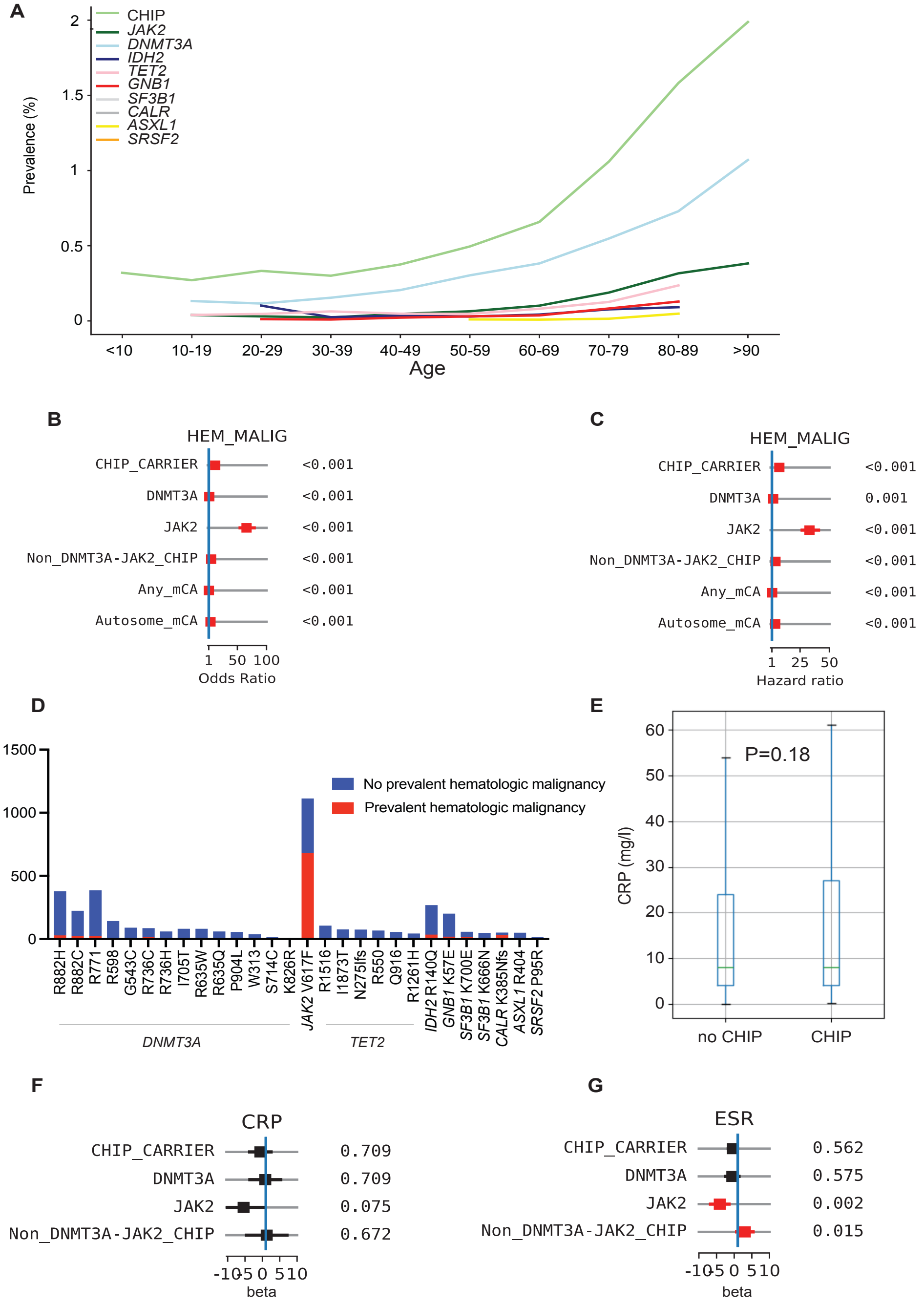

Figure S9: CHIP hotspot variants in FinnGen

(A) Age association by CHIP genes in FinnGen. (B) Association with prevalent hematologic malignancies (logistic regression). (C) Association with incident hematologic malignancies (Cox-PH). (D) Counts of variants found in participants with prevalent hematologic malignancies. (E) Association between CHIP and C-reactive protein levels (Mann-Whitney *U* test). (F) Association between CHIP subtypes and CRP levels. (G) Association between CHIP subtypes and ESR levels. C-D, F-G adjusted for age, sex, smoking, 10 principal components. E-G: Laboratory measurements are from the national registry, taken less than a year from sampling. Hematologic malignancies before of up to a year from DNA sampling are excluded.

Figure S10

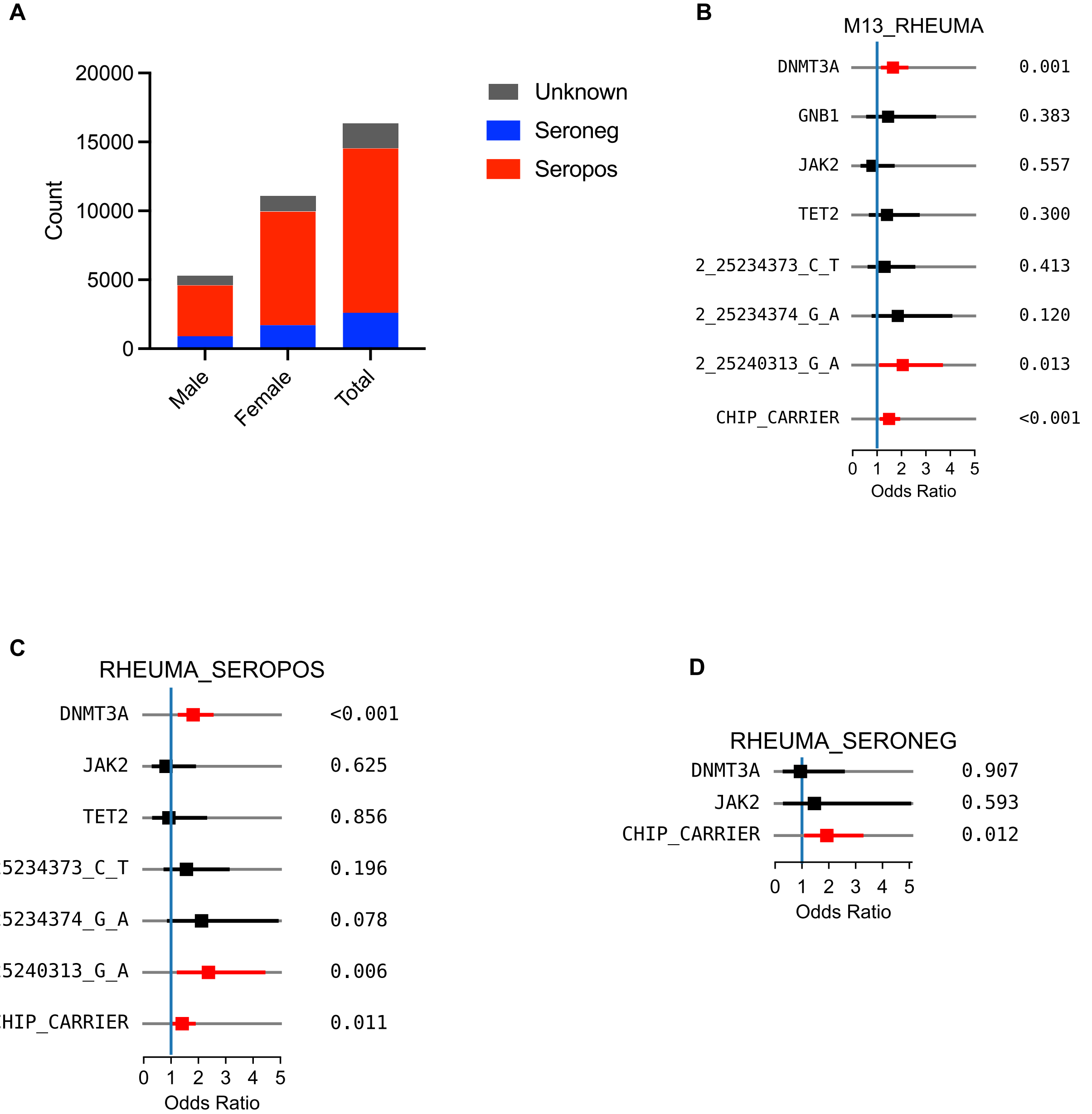

Figure S10: Association between CHIP subtypes and RA subtypes in FinnGen
(A) RA case counts in the FinnGen cohort. (B) Association of CHIP subtypes with prevalent RA. (C) Association of CHIP subtypes with prevalent seropositive RA. (D) Association of CHIP subtypes with prevalent seronegative RA. Models adjusted for age, sex, smoking and 10 principal components of ancestry, excluding prevalent hematologic malignancies.

Figure S11

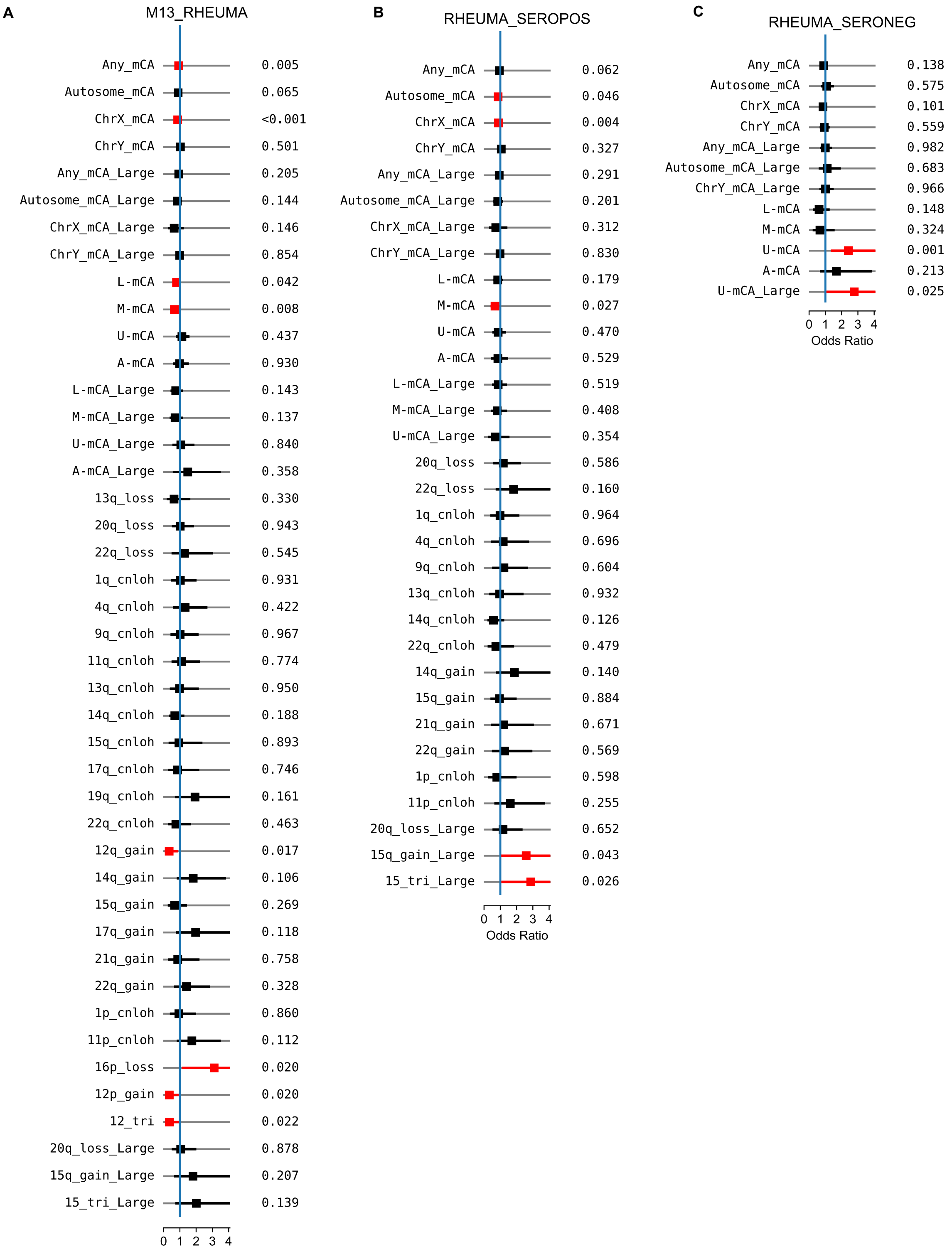

Figure S11: Associations between mCAs and RA subtypes

(A) Association between mCA subtypes and prevalent RA. (B) Association between mCA subtypes and prevalent seropositive RA. (C) Association between mCA subtypes and seronegative RA. LR models adjusted for age, sex, smoking, 10 principal components and excluding prevalent hematologic malignancies.

Figure S12

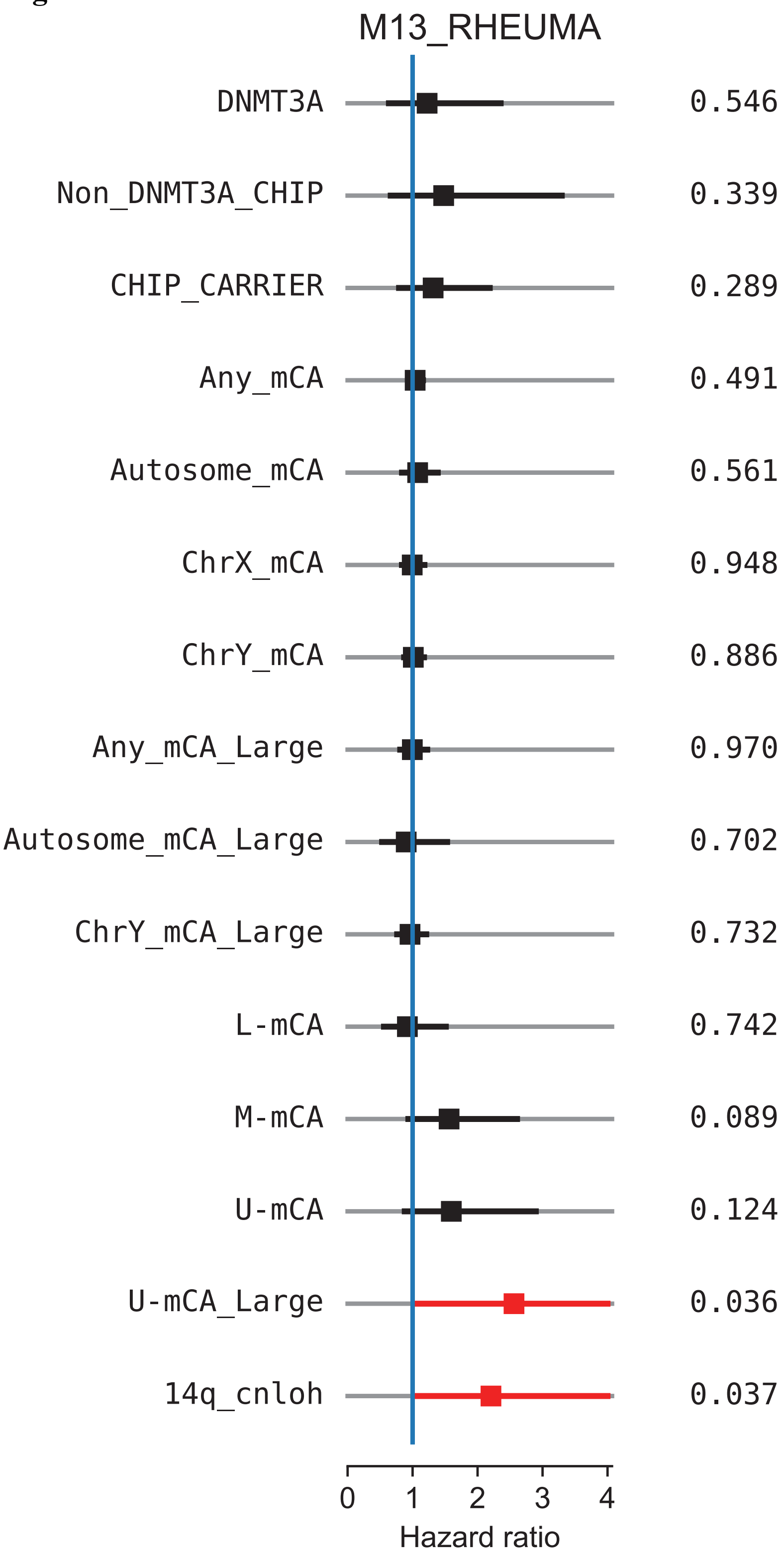

Figure S12: CH and incident RA in FinnGen.
Cox-PH models adjusted for age, sex, smoking, 10 principal components, and censoring hematologic malignancies.

Figure S13

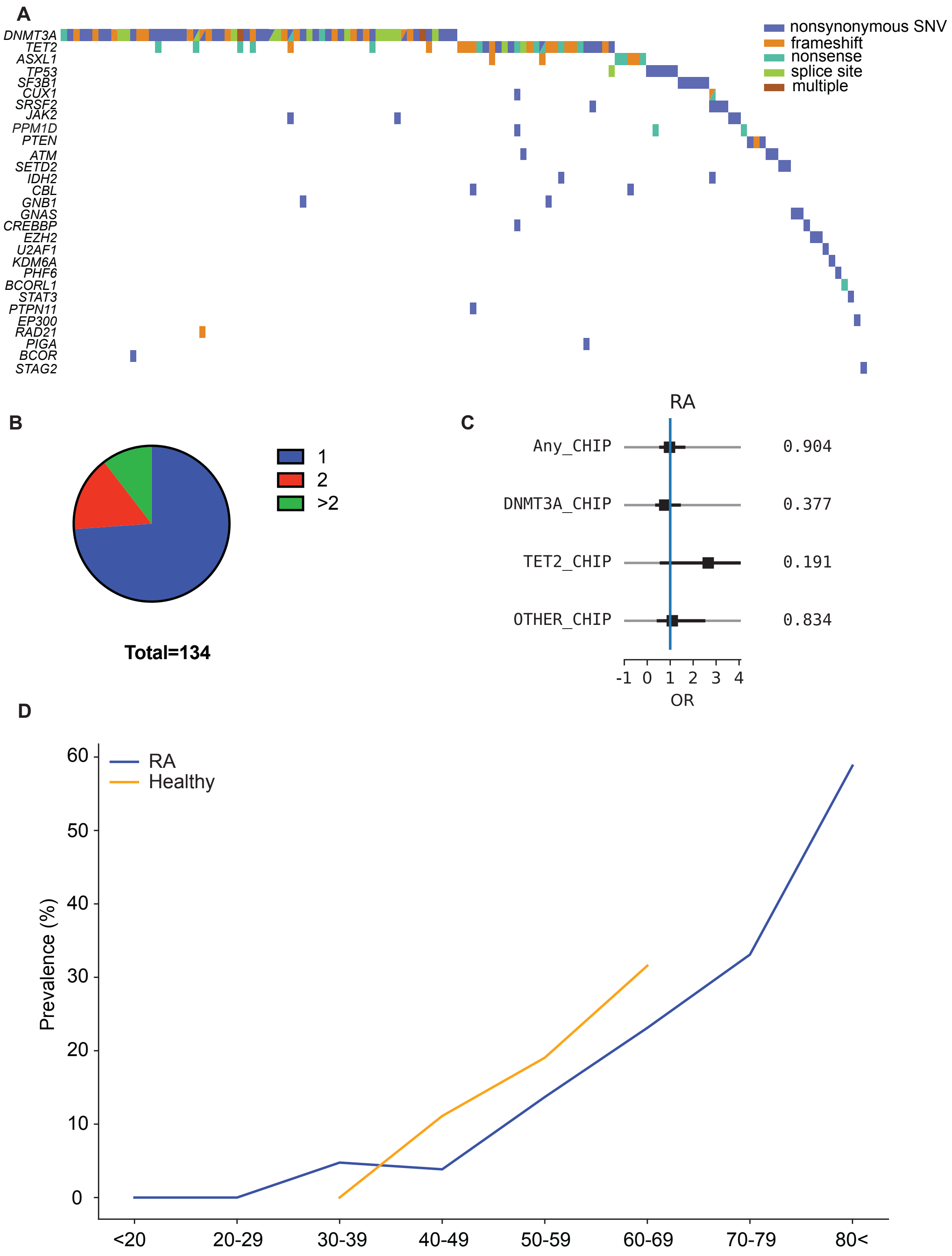

Figure S13: CHIP characteristics in RA cohort

(A) Comutation plot for RA patients. (B) Number of participants with 1, 2, or more CHIP variants. (C) CHIP status in RA vs healthy controls. Model adjusted for age and sex. (D) CHIP prevalence by age in RA patients and healthy controls.

Figure S14

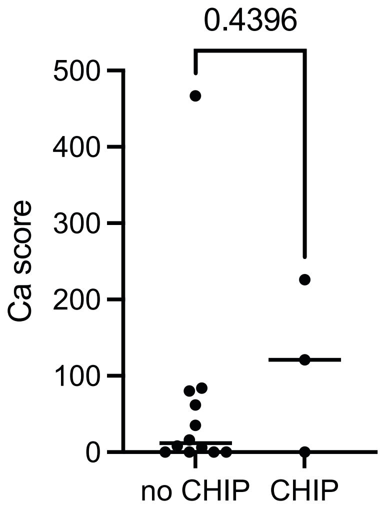

Figure S14: Ca score by CHIP status in RA patients in ERA_CVD study (N=15)

Figure S15

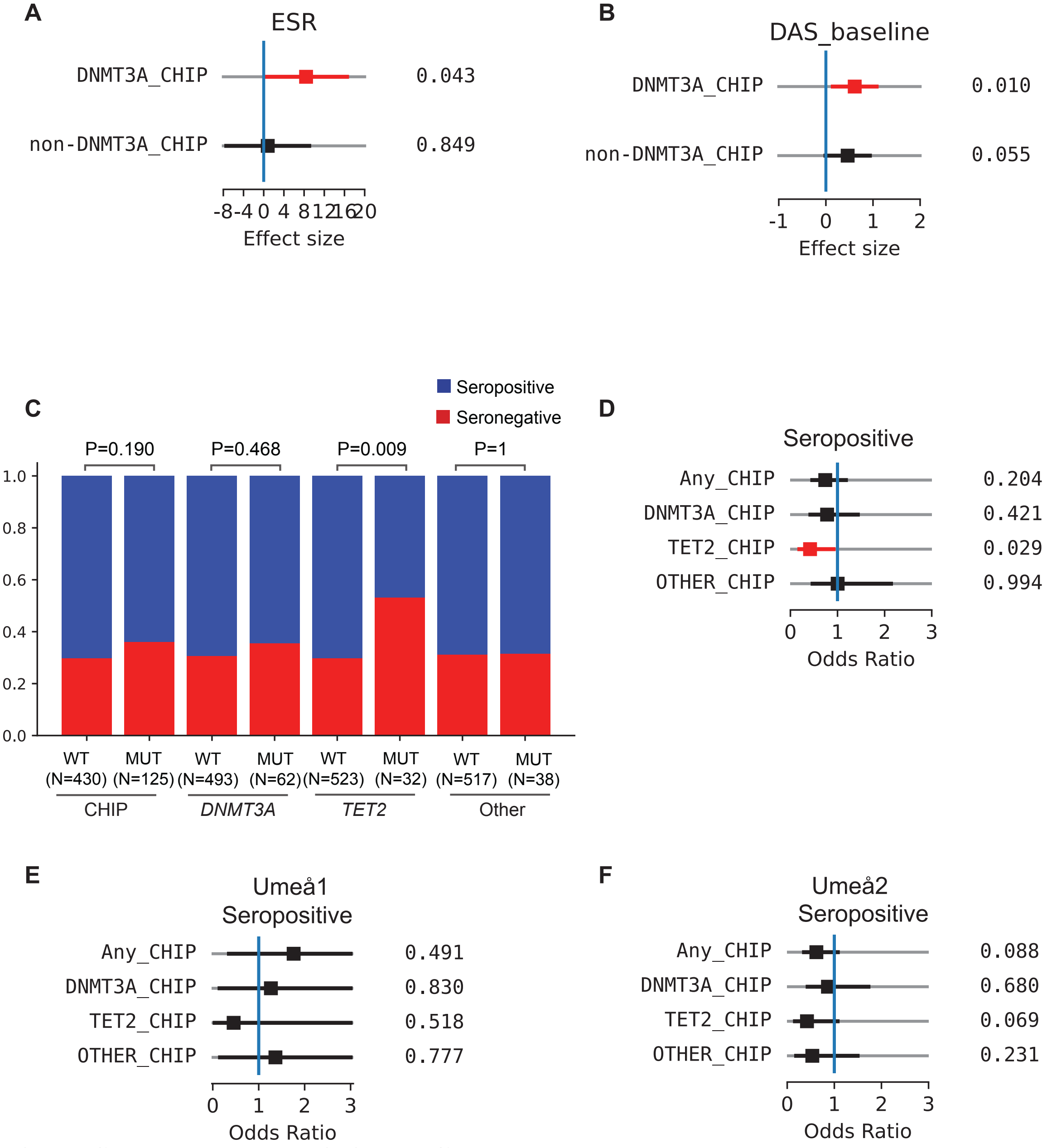

Figure S15: RA characteristics by CHIP status

(A) Multivariable association between CHIP subtypes and ESR in seropositive RA patients. (B) Multivariable association between CHIP subtypes and ESR in seropositive RA patients. A-B fitted using a least squares model adjusted for age and sex. (C) Univariable associations between CHIP subtypes and RA serostatus. P-values calculated using Fisher's exact test. (D) Multivariable model for RA serostatus by CHIP status adjusted for age, sex, smoking, ESR and CRP. (E) Multivariable model for RA serostatus in Umea1 cohort. (F) Multivariable model for RA serostatus in Umea2 cohort. D-F adjusted for age, sex, smoking.

Supplementary File S1: Tables S1-S13

Table S1: Cohorts and CH detection methods used
Table S2: CHIP candidate list
Table S3: CHIP/Lymphoid driver variants detected in FINRISK
Table S4: Lymphoid drivers candidate list
Table S5: MCAs detected in FINRISK
Table S6: SNP array derived-CHIP detection test in FINRISK
Table S7: SNP array derived CHIP filtering results and criteria
Table S8: RA patient cohort characteristics
Table S9: Panel sequencing areas
Table S10: CHIP variants detected in RA patient cohort
Table S11: Patient characteristics of newly diagnosed seronegative RA patients by *TET2* mutation status
Table S12: DRAGEN commands used in variant calling
Table S13: ICD-code based phenotype definitions

List of FinnGen authors

Full Name	Affiliation	Role 1	Role 2
Aarno Palotie	Institute for Molecular Medicine Finland (FIMM), HiLIFE, University of Helsinki, Helsinki, Finland; Broad Institute of MIT and Harvard; Massachusetts General Hospital	Steering Committee	Steering Committee
Mark Daly	Institute for Molecular Medicine Finland (FIMM), HiLIFE, University of Helsinki, Helsinki, Finland; Broad Institute of MIT and Harvard; Massachusetts General Hospital	Steering Committee	Steering Committee

Bridget Riley-Gills	Abbvie, Chicago, IL, United States	**Steering Committee**	**Pharmaceutical companies**
Howard Jacob	Abbvie, Chicago, IL, United States	**Steering Committee**	**Pharmaceutical companies**
Coralie Viollet	Astra Zeneca, Cambridge, United Kingdom	**Steering Committee**	**Pharmaceutical companies**
Slavé Petrovski	Astra Zeneca, Cambridge, United Kingdom	**Steering Committee**	**Pharmaceutical companies**
Chia-Yen Chen	Biogen, Cambridge, MA, United States	**Steering Committee**	**Pharmaceutical companies**
Sally John	Biogen, Cambridge, MA, United States	**Steering Committee**	**Pharmaceutical companies**
George Okafo	Boehringer Ingelheim, Ingelheim am Rhein, Germany	**Steering Committee**	**Pharmaceutical companies**
Robert Plenge	Bristol Myers Squibb, New York, NY, United States	**Steering Committee**	**Pharmaceutical companies**
Joseph Maranville	Bristol Myers Squibb, New York, NY, United States	**Steering Committee**	**Pharmaceutical companies**
Mark McCarthy	Genentech, San Francisco, CA, United States	**Steering Committee**	**Pharmaceutical companies**
Rion Pendergrass	Genentech, San Francisco, CA, United States	**Steering Committee**	**Pharmaceutical companies**
Jonathan Davitte	GlaxoSmithKline, Collegeville, PA, United States	**Steering Committee**	**Pharmaceutical companies**
Kirsi Auro	GlaxoSmithKline, Espoo, Finland	**Steering Committee**	**Pharmaceutical companies**
Simonne Longerich	Merck, Kenilworth, NJ, United States	**Steering Committee**	**Pharmaceutical companies**
Anders Mälarstig	Pfizer, New York, NY, United States	**Steering Committee**	**Pharmaceutical companies**

Anna Vlahiotis	Pfizer, New York, NY, United States	**Steering Committee**	**Pharmaceutical companies**
Katherine Klinger	Translational Sciences, Sanofi R&D, Framingham, MA, USA	**Steering Committee**	**Pharmaceutical companies**
Clement Chatelain	Translational Sciences, Sanofi R&D, Framingham, MA, USA	**Steering Committee**	**Pharmaceutical companies**
Jorg Blankenstein	Translational Sciences, Sanofi R&D, Framingham, MA, USA	**Steering Committee**	**Pharmaceutical companies**
Karol Estrada	Maze Therapeutics, San Francisco, CA, United States	**Steering Committee**	**Pharmaceutical companies**
Robert Graham	Maze Therapeutics, San Francisco, CA, United States	**Steering Committee**	**Pharmaceutical companies**
Dawn Waterworth	Janssen Research & Development, LLC, Spring House, PA, United States	**Steering Committee**	**Pharmaceutical companies**
Chris O´Donnell	Novartis Institutes for BioMedical Research, Cambridge, MA, United States	**Steering Committee**	**Pharmaceutical companies**
Nicole Renaud	Novartis Institutes for BioMedical Research, Cambridge, MA, United States	**Steering Committee**	**Pharmaceutical companies**
Tomi P. Mäkelä	HiLIFE, University of Helsinki, Finland, Finland	**Steering Committee**	**University of Helsinki & Biobanks**
Jaakko Kaprio	Institute for Molecular Medicine Finland (FIMM), HiLIFE, University of Helsinki, Helsinki, Finland	**Steering Committee**	**University of Helsinki & Biobanks**
Minna Ruddock	Arctic biobank / University of Oulu	**Steering Committee**	**University of Helsinki & Biobanks**

Petri Virolainen	Auria Biobank / University of Turku / Hospital District of Southwest Finland, Turku, Finland	Steering Committee	**University of Helsinki & Biobanks**
Antti Hakanen	Auria Biobank / University of Turku / Hospital District of Southwest Finland, Turku, Finland	Steering Committee	**University of Helsinki & Biobanks**
Terhi Kilpi	THL Biobank / Finnish Institute for Health and Welfare (THL), Helsinki, Finland	Steering Committee	**University of Helsinki & Biobanks**
Markus Perola	THL Biobank / Finnish Institute for Health and Welfare (THL), Helsinki, Finland	Steering Committee	**University of Helsinki & Biobanks**
Jukka Partanen	Finnish Red Cross Blood Service / Finnish Hematology Registry and Clinical Biobank, Helsinki, Finland	Steering Committee	**University of Helsinki & Biobanks**
Taneli Raivio	Helsinki Biobank / Helsinki University and Hospital District of Helsinki and Uusimaa, Helsinki	Steering Committee	**University of Helsinki & Biobanks**
Jani Tikkanen	Northern Finland Biobank Borealis / University of Oulu / Northern Ostrobothnia Hospital District, Oulu, Finland	Steering Committee	**University of Helsinki & Biobanks**
Raisa Serpi	Northern Finland Biobank Borealis / University of Oulu / Northern Ostrobothnia Hospital District, Oulu, Finland	Steering Committee	**University of Helsinki & Biobanks**
Kati Kristiansson	Finnish Clinical Biobank Tampere / University of Tampere / Pirkanmaa Hospital District, Tampere, Finland	Steering Committee	**University of Helsinki & Biobanks**

Veli-Matti Kosma	Biobank of Eastern Finland / University of Eastern Finland / Northern Savo Hospital District, Kuopio, Finland	Steering Committee	**University of Helsinki & Biobanks**
Jari Laukkanen	Central Finland Biobank / University of Jyväskylä / Central Finland Health Care District, Jyväskylä, Finland	Steering Committee	**University of Helsinki & Biobanks**
Marco Hautalahti	FINBB - Finnish biobank cooperative	Steering Committee	**University of Helsinki & Biobanks**
Outi Tuovila	Business Finland, Helsinki, Finland	Steering Committee	**Other Experts/ Non-Voting Members**
Jeffrey Waring	Abbvie, Chicago, IL, United States	Scientific Committee	**Pharmaceutical companies**
Bridget Riley-Gillis	Abbvie, Chicago, IL, United States	Scientific Committee	**Pharmaceutical companies**
Fedik Rahimov	Abbvie, Chicago, IL, United States	Scientific Committee	**Pharmaceutical companies**
Ioanna Tachmazidou	Astra Zeneca, Cambridge, United Kingdom	Scientific Committee	**Pharmaceutical companies**
Chia-Yen Chen	Biogen, Cambridge, MA, United States	Scientific Committee	**Pharmaceutical companies**
Zhihao Ding	Boehringer Ingelheim, Ingelheim am Rhein, Germany	Scientific Committee	**Pharmaceutical companies**
Marc Jung	Boehringer Ingelheim, Ingelheim am Rhein, Germany	Scientific Committee	**Pharmaceutical companies**
Hanati Tuoken	Boehringer Ingelheim, Ingelheim am Rhein, Germany	Scientific Committee	**Pharmaceutical companies**
Shameek Biswas	Bristol Myers Squibb, New York, NY, United States	Scientific Committee	**Pharmaceutical companies**

Rion Pendergrass	Genentech, San Francisco, CA, United States	Scientific Committee	**Pharmaceutical companies**
Jonathan Davitte	GlaxoSmithKline, Collegeville, PA, United States	Scientific Committee	**Pharmaceutical companies**
Neha Raghavan	Merck, Kenilworth, NJ, United States	Scientific Committee	**Pharmaceutical companies**
Adriana Huertas-Vazquez	Merck, Kenilworth, NJ, United States	Scientific Committee	**Pharmaceutical companies**
Jae-Hoon Sul	Merck, Kenilworth, NJ, United States	Scientific Committee	**Pharmaceutical companies**
Anders Mälarstig	Pfizer, New York, NY, United States	Scientific Committee	**Pharmaceutical companies**
Xinli Hu	Pfizer, New York, NY, United States	Scientific Committee	**Pharmaceutical companies**
Åsa Hedman	Pfizer, New York, NY, United States	Scientific Committee	**Pharmaceutical companies**
Katherine Klinger	Translational Sciences, Sanofi R&D, Framingham, MA, USA	Scientific Committee	**Pharmaceutical companies**
Robert Graham	Maze Therapeutics, San Francisco, CA, United States	Scientific Committee	**Pharmaceutical companies**
Dawn Waterworth	Janssen Research & Development, LLC, Spring House, PA, United States	Scientific Committee	**Pharmaceutical companies**
Nicole Renaud	Novartis Institutes for BioMedical Research, Cambridge, MA, United States	Scientific Committee	**Pharmaceutical companies**
Ma´en Obeidat	Novartis Institutes for BioMedical Research, Cambridge, MA, United States	Scientific Committee	**Pharmaceutical companies**

Jonathan Chung	Novartis Institutes for BioMedical Research, Cambridge, MA, United States	Scientific Committee	**Pharmaceutical companies**
Jonas Zierer	Novartis Institutes for BioMedical Research, Cambridge, MA, United States	Scientific Committee	**Pharmaceutical companies**
Mari Niemi	Novartis Institutes for BioMedical Research, Cambridge, MA, United States	Scientific Committee	**Pharmaceutical companies**
Samuli Ripatti	Institute for Molecular Medicine Finland (FIMM), HiLIFE, University of Helsinki, Helsinki, Finland	Scientific Committee	**University of Helsinki & Biobanks**
Johanna Schleutker	Auria Biobank / Univ. of Turku / Hospital District of Southwest Finland, Turku, Finland	Scientific Committee	**University of Helsinki & Biobanks**
Markus Perola	THL Biobank / Finnish Institute for Health and Welfare (THL), Helsinki, Finland	Scientific Committee	**University of Helsinki & Biobanks**
Mikko Arvas	Finnish Red Cross Blood Service / Finnish Hematology Registry and Clinical Biobank, Helsinki, Finland	Scientific Committee	**University of Helsinki & Biobanks**
Olli Carpén	Helsinki Biobank / Helsinki University and Hospital District of Helsinki and Uusimaa, Helsinki	Scientific Committee	**University of Helsinki & Biobanks**
Reetta Hinttala	Northern Finland Biobank Borealis / University of Oulu / Northern Ostrobothnia Hospital District, Oulu, Finland	Scientific Committee	**University of Helsinki & Biobanks**

Johannes Kettunen	Northern Finland Biobank Borealis / University of Oulu / Northern Ostrobothnia Hospital District, Oulu, Finland	Scientific Committee	**University of Helsinki & Biobanks**
Arto Mannermaa	Biobank of Eastern Finland / University of Eastern Finland / Northern Savo Hospital District, Kuopio, Finland	Scientific Committee	**University of Helsinki & Biobanks**
Katriina Aalto-Setälä	Faculty of Medicine and Health Technology, Tampere University, Tampere, Finland	Scientific Committee	**University of Helsinki & Biobanks**
Mika Kähönen	Finnish Clinical Biobank Tampere / University of Tampere / Pirkanmaa Hospital District, Tampere, Finland	Scientific Committee	**University of Helsinki & Biobanks**
Jari Laukkanen	Central Finland Biobank / University of Jyväskylä / Central Finland Health Care District, Jyväskylä, Finland	Scientific Committee	**University of Helsinki & Biobanks**
Johanna Mäkelä	FINBB - Finnish biobank cooperative	Scientific Committee	**University of Helsinki & Biobanks**
Reetta Kälviäinen	Northern Savo Hospital District, Kuopio, Finland	Clinical Groups	**Neurology Group**
Valtteri Julkunen	Northern Savo Hospital District, Kuopio, Finland	Clinical Groups	**Neurology Group**
Hilkka Soininen	Northern Savo Hospital District, Kuopio, Finland	Clinical Groups	**Neurology Group**
Anne Remes	Northern Ostrobothnia Hospital District, Oulu, Finland	Clinical Groups	**Neurology Group**

Mikko Hiltunen	University of Eastern Finland, Kuopio, Finland	Clinical Groups	**Neurology Group**
Jukka Peltola	Pirkanmaa Hospital District, Tampere, Finland	Clinical Groups	**Neurology Group**
Minna Raivio	Hospital District of Helsinki and Uusimaa, Helsinki, Finland	Clinical Groups	**Neurology Group**
Pentti Tienari	Hospital District of Helsinki and Uusimaa, Helsinki, Finland	Clinical Groups	**Neurology Group**
Juha Rinne	Hospital District of Southwest Finland, Turku, Finland	Clinical Groups	**Neurology Group**
Roosa Kallionpää	Hospital District of Southwest Finland, Turku, Finland	Clinical Groups	**Neurology Group**
Juulia Partanen	Institute for Molecular Medicine Finland, HiLIFE, University of Helsinki, Finland	Clinical Groups	**Neurology Group**
Adam Ziemann	Abbvie, Chicago, IL, United States	Clinical Groups	**Neurology Group**
Nizar Smaoui	Abbvie, Chicago, IL, United States	Clinical Groups	**Neurology Group**
Anne Lehtonen	Abbvie, Chicago, IL, United States	Clinical Groups	**Neurology Group**
Susan Eaton	Biogen, Cambridge, MA, United States	Clinical Groups	**Neurology Group**
Heiko Runz	Biogen, Cambridge, MA, United States	Clinical Groups	**Neurology Group**
Sanni Lahdenperä	Biogen, Cambridge, MA, United States	Clinical Groups	**Neurology Group**
Shameek Biswas	Bristol Myers Squibb, New York, NY, United States	Clinical Groups	**Neurology Group**
Natalie Bowers	Genentech, San Francisco, CA, United States	Clinical Groups	**Neurology Group**
Edmond Teng	Genentech, San Francisco, CA, United States	Clinical Groups	**Neurology Group**

Rion Pendergrass	Genentech, San Francisco, CA, United States	Clinical Groups	**Neurology Group**
Fanli Xu	GlaxoSmithKline, Brentford, United Kingdom	Clinical Groups	**Neurology Group**
David Pulford	GlaxoSmithKline, Stevenage, United Kingdom	Clinical Groups	**Neurology Group**
Kirsi Auro	GlaxoSmithKline, Espoo, Finland	Clinical Groups	**Neurology Group**
Laura Addis	GlaxoSmithKline, Brentford, United Kingdom	Clinical Groups	**Neurology Group**
John Eicher	GlaxoSmithKline, Brentford, United Kingdom	Clinical Groups	**Neurology Group**
Qingqin S Li	Janssen Research & Development, LLC, Titusville, NJ 08560, United States	Clinical Groups	**Neurology Group**
Karen He	Janssen Research & Development, LLC, Spring House, PA, United States	Clinical Groups	**Neurology Group**
Ekaterina Khramtsova	Janssen Research & Development, LLC, Spring House, PA, United States	Clinical Groups	**Neurology Group**
Neha Raghavan	Merck, Kenilworth, NJ, United States	Clinical Groups	**Neurology Group**
Martti Färkkilä	Hospital District of Helsinki and Uusimaa, Helsinki, Finland	Clinical Groups	**Gastroenterology Group**
Jukka Koskela	Hospital District of Helsinki and Uusimaa, Helsinki, Finland	Clinical Groups	**Gastroenterology Group**
Sampsa Pikkarainen	Hospital District of Helsinki and Uusimaa, Helsinki, Finland	Clinical Groups	**Gastroenterology Group**
Airi Jussila	Pirkanmaa Hospital District, Tampere, Finland	Clinical Groups	**Gastroenterology Group**

Katri Kaukinen	Pirkanmaa Hospital District, Tampere, Finland	Clinical Groups	**Gastroenterology Group**
Timo Blomster	Northern Ostrobothnia Hospital District, Oulu, Finland	Clinical Groups	**Gastroenterology Group**
Mikko Kiviniemi	Northern Savo Hospital District, Kuopio, Finland	Clinical Groups	**Gastroenterology Group**
Markku Voutilainen	Hospital District of Southwest Finland, Turku, Finland	Clinical Groups	**Gastroenterology Group**
Mark Daly	Institute for Molecular Medicine, Finland (FIMM), HiLIFE, University of Helsinki, Helsinki, Finland; Broad Institute of MIT and Harvard; Massachusetts General Hospital	Clinical Groups	**Gastroenterology Group**
Jeffrey Waring	Abbvie, Chicago, IL, United States	Clinical Groups	**Gastroenterology Group**
Nizar Smaoui	Abbvie, Chicago, IL, United States	Clinical Groups	**Gastroenterology Group**
Fedik Rahimov	Abbvie, Chicago, IL, United States	Clinical Groups	**Gastroenterology Group**
Anne Lehtonen	Abbvie, Chicago, IL, United States	Clinical Groups	**Gastroenterology Group**
Tim Lu	Genentech, San Francisco, CA, United States	Clinical Groups	**Gastroenterology Group**
Natalie Bowers	Genentech, San Francisco, CA, United States	Clinical Groups	**Gastroenterology Group**
Rion Pendergrass	Genentech, San Francisco, CA, United States	Clinical Groups	**Gastroenterology Group**
Linda McCarthy	GlaxoSmithKline, Brentford, United Kingdom	Clinical Groups	**Gastroenterology Group**

Amy Hart	Janssen Research & Development, LLC, Spring House, PA, United States	Clinical Groups	**Gastroenterology Group**
Meijian Guan	Janssen Research & Development, LLC, Spring House, PA, United States	Clinical Groups	**Gastroenterology Group**
Jason Miller	Merck, Kenilworth, NJ, United States	Clinical Groups	**Gastroenterology Group**
Kirsi Kalpala	Pfizer, New York, NY, United States	Clinical Groups	**Gastroenterology Group**
Melissa Miller	Pfizer, New York, NY, United States	Clinical Groups	**Gastroenterology Group**
Xinli Hu	Pfizer, New York, NY, United States	Clinical Groups	**Gastroenterology Group**
Kari Eklund	Hospital District of Helsinki and Uusimaa, Helsinki, Finland	Clinical Groups	**Rheumatology Group**
Antti Palomäki	Hospital District of Southwest Finland, Turku, Finland	Clinical Groups	**Rheumatology Group**
Pia Isomäki	Pirkanmaa Hospital District, Tampere, Finland	Clinical Groups	**Rheumatology Group**
Laura Pirilä	Hospital District of Southwest Finland, Turku, Finland	Clinical Groups	**Rheumatology Group**
Oili Kaipiainen-Seppänen	Northern Savo Hospital District, Kuopio, Finland	Clinical Groups	**Rheumatology Group**
Johanna Huhtakangas	Northern Ostrobothnia Hospital District, Oulu, Finland	Clinical Groups	**Rheumatology Group**
Nina Mars	Institute for Molecular Medicine Finland (FIMM), HiLIFE, University of Helsinki, Helsinki, Finland	Clinical Groups	**Rheumatology Group**
Jeffrey Waring	Abbvie, Chicago, IL, United States	Clinical Groups	**Rheumatology Group**

Fedik Rahimov	Abbvie, Chicago, IL, United States	Clinical Groups	**Rheumatology Group**
Apinya Lertratanakul	Abbvie, Chicago, IL, United States	Clinical Groups	**Rheumatology Group**
Nizar Smaoui	Abbvie, Chicago, IL, United States	Clinical Groups	**Rheumatology Group**
Anne Lehtonen	Abbvie, Chicago, IL, United States	Clinical Groups	**Rheumatology Group**
Coralie Viollet	AstraZeneca, Cambridge, United Kingdom	Clinical Groups	**Rheumatology Group**
Marla Hochfeld	Bristol Myers Squibb, New York, NY, United States	Clinical Groups	**Rheumatology Group**
Natalie Bowers	Genentech, San Francisco, CA, United States	Clinical Groups	**Rheumatology Group**
Rion Pendergrass	Genentech, San Francisco, CA, United States	Clinical Groups	**Rheumatology Group**
Jorge Esparza Gordillo	GlaxoSmithKline, Brentford, United Kingdom	Clinical Groups	**Rheumatology Group**
Kirsi Auro	GlaxoSmithKline, Espoo, Finland	Clinical Groups	**Rheumatology Group**
Dawn Waterworth	Janssen Research & Development, LLC, Spring House, PA, United States	Clinical Groups	**Rheumatology Group**
Fabiana Farias	Merck, Kenilworth, NJ, United States	Clinical Groups	**Rheumatology Group**
Kirsi Kalpala	Pfizer, New York, NY, United States	Clinical Groups	**Rheumatology Group**
Nan Bing	Pfizer, New York, NY, United States	Clinical Groups	**Rheumatology Group**
Xinli Hu	Pfizer, New York, NY, United States	Clinical Groups	**Rheumatology Group**
Tarja Laitinen	Pirkanmaa Hospital District, Tampere, Finland	Clinical Groups	**Pulmonology Group**
Margit Pelkonen	Northern Savo Hospital District, Kuopio, Finland	Clinical Groups	**Pulmonology Group**

Paula Kauppi	Hospital District of Helsinki and Uusimaa, Helsinki, Finland	Clinical Groups	**Pulmonology Group**
Hannu Kankaanranta	University of Gothenburg, Gothenburg, Sweden/ Seinäjoki Central Hospital, Seinäjoki, Finland/ Tampere University, Tampere, Finland	Clinical Groups	**Pulmonology Group**
Terttu Harju	Northern Ostrobothnia Hospital District, Oulu, Finland	Clinical Groups	**Pulmonology Group**
Riitta Lahesmaa	Hospital District of Southwest Finland, Turku, Finland	Clinical Groups	**Pulmonology Group**
Nizar Smaoui	Abbvie, Chicago, IL, United States	Clinical Groups	**Pulmonology Group**
Coralie Viollet	AstraZeneca, Cambridge, United Kingdom	Clinical Groups	**Pulmonology Group**
Susan Eaton	Biogen, Cambridge, MA, United States	Clinical Groups	**Pulmonology Group**
Hubert Chen	Genentech, San Francisco, CA, United States	Clinical Groups	**Pulmonology Group**
Rion Pendergrass	Genentech, San Francisco, CA, United States	Clinical Groups	**Pulmonology Group**
Natalie Bowers	Genentech, San Francisco, CA, United States	Clinical Groups	**Pulmonology Group**
Joanna Betts	GlaxoSmithKline, Brentford, United Kingdom	Clinical Groups	**Pulmonology Group**
Kirsi Auro	GlaxoSmithKline, Espoo, Finland	Clinical Groups	**Pulmonology Group**
Rajashree Mishra	GlaxoSmithKline, Brentford, United Kingdom	Clinical Groups	**Pulmonology Group**
Majd Mouded	Novartis, Basel, Switzerland	Clinical Groups	**Pulmonology Group**
Debby Ngo	Novartis, Basel, Switzerland	Clinical Groups	**Pulmonology Group**

Teemu Niiranen	Finnish Institute for Health and Welfare (THL), Helsinki, Finland	**Clinical Groups**	**Cardiometabolic Diseases Group**
Felix Vaura	Finnish Institute for Health and Welfare (THL), Helsinki, Finland	**Clinical Groups**	**Cardiometabolic Diseases Group**
Veikko Salomaa	Finnish Institute for Health and Welfare (THL), Helsinki, Finland	**Clinical Groups**	**Cardiometabolic Diseases Group**
Kaj Metsärinne	Hospital District of Southwest Finland, Turku, Finland	**Clinical Groups**	**Cardiometabolic Diseases Group**
Jenni Aittokallio	Hospital District of Southwest Finland, Turku, Finland	**Clinical Groups**	**Cardiometabolic Diseases Group**
Mika Kähönen	Pirkanmaa Hospital District, Tampere, Finland	**Clinical Groups**	**Cardiometabolic Diseases Group**
Jussi Hernesniemi	Pirkanmaa Hospital District, Tampere, Finland	**Clinical Groups**	**Cardiometabolic Diseases Group**
Daniel Gordin	Hospital District of Helsinki and Uusimaa, Helsinki, Finland	**Clinical Groups**	**Cardiometabolic Diseases Group**
Juha Sinisalo	Hospital District of Helsinki and Uusimaa, Helsinki, Finland	**Clinical Groups**	**Cardiometabolic Diseases Group**
Marja-Riitta Taskinen	Hospital District of Helsinki and Uusimaa, Helsinki, Finland	**Clinical Groups**	**Cardiometabolic Diseases Group**
Tiinamaija Tuomi	Hospital District of Helsinki and Uusimaa, Helsinki, Finland	**Clinical Groups**	**Cardiometabolic Diseases Group**

Timo Hiltunen	Hospital District of Helsinki and Uusimaa, Helsinki, Finland	Clinical Groups	**Cardiometabolic Diseases Group**
Jari Laukkanen	Central Finland Health Care District, Jyväskylä, Finland	Clinical Groups	**Cardiometabolic Diseases Group**
Amanda Elliott	Institute for Molecular Medicine Finland (FIMM), HiLIFE, University of Helsinki, Helsinki, Finland; Broad Institute, Cambridge, MA, USA and Massachusetts General Hospital, Boston, MA, USA	Clinical Groups	**Cardiometabolic Diseases Group**
Mary Pat Reeve	Institute for Molecular Medicine Finland (FIMM), HiLIFE, University of Helsinki, Helsinki, Finland	Clinical Groups	**Cardiometabolic Diseases Group**
Sanni Ruotsalainen	Institute for Molecular Medicine Finland (FIMM), HiLIFE, University of Helsinki, Helsinki, Finland	Clinical Groups	**Cardiometabolic Diseases Group**
Dirk Paul	Astra Zeneca, Cambridge, United Kingdom	Clinical Groups	**Cardiometabolic Diseases Group**
Natalie Bowers	Genentech, San Francisco, CA, United States	Clinical Groups	**Cardiometabolic Diseases Group**
Rion Pendergrass	Genentech, San Francisco, CA, United States	Clinical Groups	**Cardiometabolic Diseases Group**
Audrey Chu	GlaxoSmithKline, Brentford, United Kingdom	Clinical Groups	**Cardiometabolic Diseases Group**

Kirsi Auro	GlaxoSmithKline, Espoo, Finland	Clinical Groups	**Cardiometabolic Diseases Group**
Dermot Reilly	Janssen Research & Development, LLC, Boston, MA, United States	Clinical Groups	**Cardiometabolic Diseases Group**
Mike Mendelson	Novartis, Boston, MA, United States	Clinical Groups	**Cardiometabolic Diseases Group**
Jaakko Parkkinen	Pfizer, New York, NY, United States	Clinical Groups	**Cardiometabolic Diseases Group**
Melissa Miller	Pfizer, New York, NY, United States	Clinical Groups	**Cardiometabolic Diseases Group**
Tuomo Meretoja	Department of Breast Surgery, Helsinki University Hospital Comprehensive Cancer Center and University of Helsinki, Helsinki, Finland	Clinical Groups	**Oncology Group**
Heikki Joensuu	Department of Oncology, Helsinki University Hospital Comprehensive Cancer Center and University of Helsinki, Helsinki, Finland	Clinical Groups	**Oncology Group**
Olli Carpén	Hospital District of Helsinki and Uusimaa, Helsinki, Finland	Clinical Groups	**Oncology Group**
Johanna Mattson	Hospital District of Helsinki and Uusimaa, Helsinki, Finland	Clinical Groups	**Oncology Group**
Eveliina Salminen	Hospital District of Helsinki and Uusimaa, Helsinki, Finland	Clinical Groups	**Oncology Group**

Annika Auranen	Pirkanmaa Hospital District , Tampere, Finland	Clinical Groups	**Oncology Group**
Peeter Karihtala	Department of Oncology, Helsinki University Hospital Comprehensive Cancer Center and University of Helsinki, Helsinki, Finland	Clinical Groups	**Oncology Group**
Päivi Auvinen	Northern Savo Hospital District, Kuopio, Finland	Clinical Groups	**Oncology Group**
Klaus Elenius	Hospital District of Southwest Finland, Turku, Finland	Clinical Groups	**Oncology Group**
Johanna Schleutker	Hospital District of Southwest Finland, Turku, Finland	Clinical Groups	**Oncology Group**
Esa Pitkänen	Institute for Molecular Medicine Finland (FIMM), HiLIFE, University of Helsinki, Helsinki, Finland	Clinical Groups	**Oncology Group**
Nina Mars	Institute for Molecular Medicine Finland (FIMM), HiLIFE, University of Helsinki, Helsinki, Finland	Clinical Groups	**Oncology Group**
Mark Daly	Institute for Molecular Medicine Finland (FIMM), HiLIFE, University of Helsinki, Helsinki, Finland; Broad Institute of MIT and Harvard; Massachusetts General Hospital	Clinical Groups	**Oncology Group**
Relja Popovic	Abbvie, Chicago, IL, United States	Clinical Groups	**Oncology Group**
Jeffrey Waring	Abbvie, Chicago, IL, United States	Clinical Groups	**Oncology Group**
Bridget Riley-Gillis	Abbvie, Chicago, IL, United States	Clinical Groups	**Oncology Group**
Anne Lehtonen	Abbvie, Chicago, IL, United States	Clinical Groups	**Oncology Group**

Margarete Fabre	AstraZeneca, Cambridge, United Kingdom	Clinical Groups	**Oncology Group**
Jennifer Schutzman	Genentech, San Francisco, CA, United States	Clinical Groups	**Oncology Group**
Natalie Bowers	Genentech, San Francisco, CA, United States	Clinical Groups	**Oncology Group**
Rion Pendergrass	Genentech, San Francisco, CA, United States	Clinical Groups	**Oncology Group**
Diptee Kulkarni	GlaxoSmithKline, Brentford, United Kingdom	Clinical Groups	**Oncology Group**
Kirsi Auro	GlaxoSmithKline, Espoo, Finland	Clinical Groups	**Oncology Group**
Alessandro Porello	Janssen Research & Development, LLC, Spring House, PA, United States	Clinical Groups	**Oncology Group**
Andrey Loboda	Merck, Kenilworth, NJ, United States	Clinical Groups	**Oncology Group**
Heli Lehtonen	Pfizer, New York, NY, United States	Clinical Groups	**Oncology Group**
Stefan McDonough	Pfizer, New York, NY, United States	Clinical Groups	**Oncology Group**
Sauli Vuoti	Janssen-Cilag Oy, Espoo, Finland	Clinical Groups	**Oncology Group**
Kai Kaarniranta	Northern Savo Hospital District, Kuopio, Finland; Department of Molecular Genetics, University of Lodz, Lodz, Poland	Clinical Groups	**Opthalmology Group**
Joni A Turunen	Helsinki University Hospital and University of Helsinki, Helsinki, Finland; Eye Genetics Group, Folkhälsan Research Center, Helsinki, Finland	Clinical Groups	**Opthalmology Group**
Terhi Ollila	Hospital District of Helsinki and Uusimaa, Helsinki, Finland	Clinical Groups	**Opthalmology Group**

Hannu Uusitalo	Pirkanmaa Hospital District, Tampere, Finland	Clinical Groups	**Opthalmology Group**
Juha Karjalainen	Institute for Molecular Medicine Finland (FIMM), HiLIFE, University of Helsinki, Helsinki, Finland	Clinical Groups	**Opthalmology Group**
Esa Pitkänen	Institute for Molecular Medicine Finland (FIMM), HiLIFE, University of Helsinki, Helsinki, Finland	Clinical Groups	**Opthalmology Group**
Mengzhen Liu	Abbvie, Chicago, IL, United States	Clinical Groups	**Opthalmology Group**
Heiko Runz	Biogen, Cambridge, MA, United States	Clinical Groups	**Opthalmology Group**
Stephanie Loomis	Biogen, Cambridge, MA, United States	Clinical Groups	**Opthalmology Group**
Erich Strauss	Genentech, San Francisco, CA, United States	Clinical Groups	**Opthalmology Group**
Natalie Bowers	Genentech, San Francisco, CA, United States	Clinical Groups	**Opthalmology Group**
Hao Chen	Genentech, San Francisco, CA, United States	Clinical Groups	**Opthalmology Group**
Rion Pendergrass	Genentech, San Francisco, CA, United States	Clinical Groups	**Opthalmology Group**
Kaisa Tasanen	Northern Ostrobothnia Hospital District, Oulu, Finland	Clinical Groups	**Dermatology Group**
Laura Huilaja	Northern Ostrobothnia Hospital District, Oulu, Finland	Clinical Groups	**Dermatology Group**
Katariina Hannula-Jouppi	Hospital District of Helsinki and Uusimaa, Helsinki, Finland	Clinical Groups	**Dermatology Group**
Teea Salmi	Pirkanmaa Hospital District, Tampere, Finland	Clinical Groups	**Dermatology Group**

Sirkku Peltonen	Hospital District of Southwest Finland, Turku, Finland	Clinical Groups	**Dermatology Group**
Leena Koulu	Hospital District of Southwest Finland, Turku, Finland	Clinical Groups	**Dermatology Group**
Nizar Smaoui	Abbvie, Chicago, IL, United States	Clinical Groups	**Dermatology Group**
Fedik Rahimov	Abbvie, Chicago, IL, United States	Clinical Groups	**Dermatology Group**
Anne Lehtonen	Abbvie, Chicago, IL, United States	Clinical Groups	**Dermatology Group**
David Choy	Genentech, San Francisco, CA, United States	Clinical Groups	**Dermatology Group**
Rion Pendergrass	Genentech, San Francisco, CA, United States	Clinical Groups	**Dermatology Group**
Dawn Waterworth	Janssen Research & Development, LLC, Spring House, PA, United States	Clinical Groups	**Dermatology Group**
Kirsi Kalpala	Pfizer, New York, NY, United States	Clinical Groups	**Dermatology Group**
Ying Wu	Pfizer, New York, NY, United States	Clinical Groups	**Dermatology Group**
Pirkko Pussinen	Hospital District of Helsinki and Uusimaa, Helsinki, Finland	Clinical Groups	**Odontology Group**
Aino Salminen	Hospital District of Helsinki and Uusimaa, Helsinki, Finland	Clinical Groups	**Odontology Group**
Tuula Salo	Hospital District of Helsinki and Uusimaa, Helsinki, Finland	Clinical Groups	**Odontology Group**
David Rice	Hospital District of Helsinki and Uusimaa, Helsinki, Finland	Clinical Groups	**Odontology Group**
Pekka Nieminen	Hospital District of Helsinki and Uusimaa, Helsinki, Finland	Clinical Groups	**Odontology Group**
Ulla Palotie	Hospital District of Helsinki and Uusimaa, Helsinki, Finland	Clinical Groups	**Odontology Group**

Maria Siponen	Northern Savo Hospital District, Kuopio, Finland	Clinical Groups	**Odontology Group**
Liisa Suominen	Northern Savo Hospital District, Kuopio, Finland	Clinical Groups	**Odontology Group**
Päivi Mäntylä	Northern Savo Hospital District, Kuopio, Finland	Clinical Groups	**Odontology Group**
Ulvi Gursoy	Hospital District of Southwest Finland, Turku, Finland	Clinical Groups	**Odontology Group**
Vuokko Anttonen	Northern Ostrobothnia Hospital District, Oulu, Finland	Clinical Groups	**Odontology Group**
Kirsi Sipilä	Research Unit of Oral Health Sciences Faculty of Medicine, University of Oulu, Oulu, Finland; Medical Research Center, Oulu, Oulu University Hospital and University of Oulu, Oulu, Finland	Clinical Groups	**Odontology Group**
Rion Pendergrass	Genentech, San Francisco, CA, United States	Clinical Groups	**Odontology Group**
Hannele Laivuori	Institute for Molecular Medicine Finland (FIMM), HiLIFE, University of Helsinki, Helsinki, Finland	Clinical Groups	**Women's Health and Reproduction Group**
Venla Kurra	Pirkanmaa Hospital District, Tampere, Finland	Clinical Groups	**Women's Health and Reproduction Group**
Laura Kotaniemi-Talonen	Pirkanmaa Hospital District, Tampere, Finland	Clinical Groups	**Women's Health and Reproduction Group**
Oskari Heikinheimo	Hospital District of Helsinki and Uusimaa, Helsinki, Finland	Clinical Groups	**Women's Health and Reproduction Group**

Ilkka Kalliala	Hospital District of Helsinki and Uusimaa, Helsinki, Finland	Clinical Groups	**Women's Health and Reproduction Group**
Lauri Aaltonen	Hospital District of Helsinki and Uusimaa, Helsinki, Finland	Clinical Groups	**Women's Health and Reproduction Group**
Varpu Jokimaa	Hospital District of Southwest Finland, Turku, Finland	Clinical Groups	**Women's Health and Reproduction Group**
Johannes Kettunen	Northern Ostrobothnia Hospital District, Oulu, Finland	Clinical Groups	**Women's Health and Reproduction Group**
Marja Vääräsmäki	Northern Ostrobothnia Hospital District, Oulu, Finland	Clinical Groups	**Women's Health and Reproduction Group**
Outi Uimari	Northern Ostrobothnia Hospital District, Oulu, Finland	Clinical Groups	**Women's Health and Reproduction Group**
Laure Morin-Papunen	Northern Ostrobothnia Hospital District, Oulu, Finland	Clinical Groups	**Women's Health and Reproduction Group**
Maarit Niinimäki	Northern Ostrobothnia Hospital District, Oulu, Finland	Clinical Groups	**Women's Health and Reproduction Group**
Terhi Piltonen	Northern Ostrobothnia Hospital District, Oulu, Finland	Clinical Groups	**Women's Health and Reproduction Group**
Katja Kivinen	Institute for Molecular Medicine Finland (FIMM), HiLIFE, University of Helsinki, Helsinki, Finland	Clinical Groups	**Women's Health and Reproduction Group**
Elisabeth Widen	Institute for Molecular Medicine Finland (FIMM), HiLIFE, University of Helsinki, Helsinki, Finland	Clinical Groups	**Women's Health and Reproduction Group**

Taru Tukiainen	Institute for Molecular Medicine Finland (FIMM), HiLIFE, University of Helsinki, Helsinki, Finland	**Clinical Groups**	**Women's Health and Reproduction Group**
Mary Pat Reeve	Institute for Molecular Medicine Finland (FIMM), HiLIFE, University of Helsinki, Helsinki, Finland	**Clinical Groups**	**Women's Health and Reproduction Group**
Mark Daly	Institute for Molecular Medicine Finland (FIMM), HiLIFE, University of Helsinki, Helsinki, Finland; Broad Institute of MIT and Harvard; Massachusetts General Hospital	**Clinical Groups**	**Women's Health and Reproduction Group**
Niko Välimäki	University of Helsinki, Helsinki, Finland	**Clinical Groups**	**Women's Health and Reproduction Group**
Eija Laakkonen	University of Jyväskylä, Jyväskylä, Finland	**Clinical Groups**	**Women's Health and Reproduction Group**
Jaakko Tyrmi	University of Oulu, Oulu, Finland / University of Tampere, Tampere, Finland	**Clinical Groups**	**Women's Health and Reproduction Group**
Heidi Silven	University of Oulu, Oulu, Finland	**Clinical Groups**	**Women's Health and Reproduction Group**
Eeva Sliz	University of Oulu, Oulu, Finland	**Clinical Groups**	**Women's Health and Reproduction Group**
Riikka Arffman	University of Oulu, Oulu, Finland	**Clinical Groups**	**Women's Health and Reproduction Group**

Susanna Savukoski	University of Oulu, Oulu, Finland	Clinical Groups	**Women's Health and Reproduction Group**
Triin Laisk	Estonian biobank, Tartu, Estonia	Clinical Groups	**Women's Health and Reproduction Group**
Natalia Pujol	Estonian biobank, Tartu, Estonia	Clinical Groups	**Women's Health and Reproduction Group**
Mengzhen Liu	Abbvie, Chicago, IL, United States	Clinical Groups	**Women's Health and Reproduction Group**
Bridget Riley-Gillis	Abbvie, Chicago, IL, United States	Clinical Groups	**Women's Health and Reproduction Group**
Rion Pendergrass	Genentech, San Francisco, CA, United States	Clinical Groups	**Women's Health and Reproduction Group**
Janet Kumar	GlaxoSmithKline, Collegeville, PA, United States	Clinical Groups	**Women's Health and Reproduction Group**
Kirsi Auro	GlaxoSmithKline, Espoo, Finland	Clinical Groups	**Women's Health and Reproduction Group**
Iiris Hovatta	University of Helsinki, Finland	Clinical Groups	**Depression group**
Chia-Yen Chen	Biogen, Cambridge, MA, United States	Clinical Groups	**Depression group**
Erkki Isometsä	Hospital District of Helsinki and Uusimaa, Helsinki, Finland	Clinical Groups	**Depression group**
Hanna Ollila	Institute for Molecular Medicine Finland (FIMM), HiLIFE, University of Helsinki, Helsinki, Finland	Clinical Groups	**Depression group**

Jaana Suvisaari	Finnish Institute for Health and Welfare (THL), Helsinki, Finland	Clinical Groups	**Depression group**
Antti Mäkitie	Department of Otorhinolaryngology - Head and Neck Surgery, University of Helsinki and Helsinki University Hospital, Helsinki, Finland	Clinical Groups	**ENT (ear, nose and throath) Group**
Argyro Bizaki-Vallaskangas	Pirkanmaa Hospital District, Tampere, Finland	Clinical Groups	**ENT (ear, nose and throath) Group**
Sanna Toppila-Salmi	University of Eastern Finland and Kuopio University Hospital, Department of Otorhinolaryngology, Kuopio, Finland and Department of Allergy, Helsinki University Hospital and University of Helsinki, Finland	Clinical Groups	**ENT (ear, nose and throath) Group**
Tytti Willberg	Hospital District of Southwest Finland, Turku, Finland	Clinical Groups	**ENT (ear, nose and throath) Group**
Elmo Saarentaus	Institute for Molecular Medicine Finland (FIMM), HiLIFE, University of Helsinki, Helsinki, Finland	Clinical Groups	**ENT (ear, nose and throath) Group**
Antti Aarnisalo	Hospital District of Helsinki and Uusimaa, Helsinki, Finland	Clinical Groups	**ENT (ear, nose and throath) Group**
Eveliina Salminen	Hospital District of Helsinki and Uusimaa, Helsinki, Finland	Clinical Groups	**ENT (ear, nose and throath) Group**
Elisa Rahikkala	Northern Ostrobothnia Hospital District, Oulu, Finland	Clinical Groups	**ENT (ear, nose and throath) Group**

Johannes Kettunen	Northern Ostrobothnia Hospital District, Oulu, Finland	**Clinical Groups**	**ENT (ear, nose and throath) Group**
Kristiina Aittomäki	Department of Medical Genetics, Helsinki University Central Hospital, Helsinki, Finland	**Clinical Groups**	**POI (premature ovarian failure) Group**
Fredrik Åberg	Transplantation and Liver Surgery Clinic, Helsinki University Hospital, Helsinki University, Helsinki, Finland	**Clinical Groups**	**LiverScore Group**
Mitja Kurki	Institute for Molecular Medicine Finland (FIMM), HiLIFE, University of Helsinki, Helsinki, Finland; Broad Institute, Cambridge, MA, United States	**FinnGen Analysis working group**	**FinnGen Analysis working group**
Samuli Ripatti	Institute for Molecular Medicine Finland (FIMM), HiLIFE, University of Helsinki, Helsinki, Finland	**FinnGen Analysis working group**	**FinnGen Analysis working group**
Mark Daly	Institute for Molecular Medicine, Finland (FIMM), HiLIFE, University of Helsinki, Helsinki, Finland; Broad Institute of MIT and Harvard; Massachusetts General Hospital	**FinnGen Analysis working group**	**FinnGen Analysis working group**
Juha Karjalainen	Institute for Molecular Medicine Finland (FIMM), HiLIFE, University of Helsinki, Helsinki, Finland	**FinnGen Analysis working group**	**FinnGen Analysis working group**

Aki Havulinna	Institute for Molecular Medicine Finland (FIMM), HiLIFE, University of Helsinki, Helsinki, Finland; Finnish Institute for Health and Welfare (THL), Helsinki, Finland	**FinnGen Analysis working group**	**FinnGen Analysis working group**
Juha Mehtonen	Institute for Molecular Medicine Finland (FIMM), HiLIFE, University of Helsinki, Helsinki, Finland	**FinnGen Analysis working group**	**FinnGen Analysis working group**
Priit Palta	Institute for Molecular Medicine Finland (FIMM), HiLIFE, University of Helsinki, Helsinki, Finland	**FinnGen Analysis working group**	**FinnGen Analysis working group**
Shabbeer Hassan	Institute for Molecular Medicine Finland (FIMM), HiLIFE, University of Helsinki, Helsinki, Finland	**FinnGen Analysis working group**	**FinnGen Analysis working group**
Pietro Della Briotta Parolo	Institute for Molecular Medicine Finland (FIMM), HiLIFE, University of Helsinki, Helsinki, Finland	**FinnGen Analysis working group**	**FinnGen Analysis working group**
Wei Zhou	Broad Institute, Cambridge, MA, United States	**FinnGen Analysis working group**	**FinnGen Analysis working group**
Mutaamba Maasha	Broad Institute, Cambridge, MA, United States	**FinnGen Analysis working group**	**FinnGen Analysis working group**
Shabbeer Hassan	Institute for Molecular Medicine Finland (FIMM), HiLIFE, University of Helsinki, Helsinki, Finland	**FinnGen Analysis working group**	**FinnGen Analysis working group**
Susanna Lemmelä	Institute for Molecular Medicine Finland (FIMM), HiLIFE, University of Helsinki, Helsinki, Finland	**FinnGen Analysis working group**	**FinnGen Analysis working group**

Manuel Rivas	University of Stanford, Stanford, CA, United States	FinnGen Analysis working group	FinnGen Analysis working group
Aarno Palotie	Institute for Molecular Medicine Finland (FIMM), HiLIFE, University of Helsinki, Helsinki, Finland	FinnGen Analysis working group	FinnGen Analysis working group
Aoxing Liu	Institute for Molecular Medicine Finland (FIMM), HiLIFE, University of Helsinki, Helsinki, Finland	FinnGen Analysis working group	FinnGen Analysis working group
Arto Lehisto	Institute for Molecular Medicine Finland (FIMM), HiLIFE, University of Helsinki, Helsinki, Finland	FinnGen Analysis working group	FinnGen Analysis working group
Andrea Ganna	Institute for Molecular Medicine Finland (FIMM), HiLIFE, University of Helsinki, Helsinki, Finland	FinnGen Analysis working group	FinnGen Analysis working group
Vincent Llorens	Institute for Molecular Medicine Finland (FIMM), HiLIFE, University of Helsinki, Helsinki, Finland	FinnGen Analysis working group	FinnGen Analysis working group
Hannele Laivuori	Institute for Molecular Medicine Finland (FIMM), HiLIFE, University of Helsinki, Helsinki, Finland	FinnGen Analysis working group	FinnGen Analysis working group
Taru Tukiainen	Institute for Molecular Medicine Finland (FIMM), HiLIFE, University of Helsinki, Helsinki, Finland	FinnGen Analysis working group	FinnGen Analysis working group
Mary Pat Reeve	Institute for Molecular Medicine Finland (FIMM), HiLIFE, University of Helsinki, Helsinki, Finland	FinnGen Analysis working group	FinnGen Analysis working group

Henrike Heyne	Institute for Molecular Medicine Finland (FIMM), HiLIFE, University of Helsinki, Helsinki, Finland	FinnGen Analysis working group	FinnGen Analysis working group
Nina Mars	Institute for Molecular Medicine Finland (FIMM), HiLIFE, University of Helsinki, Helsinki, Finland	FinnGen Analysis working group	FinnGen Analysis working group
Joel Rämö	Institute for Molecular Medicine Finland (FIMM), HiLIFE, University of Helsinki, Helsinki, Finland	FinnGen Analysis working group	FinnGen Analysis working group
Elmo Saarentaus	Institute for Molecular Medicine Finland (FIMM), HiLIFE, University of Helsinki, Helsinki, Finland	FinnGen Analysis working group	FinnGen Analysis working group
Hanna Ollila	Institute for Molecular Medicine Finland (FIMM), HiLIFE, University of Helsinki, Helsinki, Finland	FinnGen Analysis working group	FinnGen Analysis working group
Satu Strausz	Institute for Molecular Medicine Finland (FIMM), HiLIFE, University of Helsinki, Helsinki, Finland	FinnGen Analysis working group	FinnGen Analysis working group
Tuula Palotie	University of Helsinki and Hospital District of Helsinki and Uusimaa, Helsinki, Finland	FinnGen Analysis working group	FinnGen Analysis working group
Kimmo Palin	University of Helsinki, Helsinki, Finland	FinnGen Analysis working group	FinnGen Analysis working group
Javier Garcia-Tabuenca	University of Tampere, Tampere, Finland	FinnGen Analysis working group	FinnGen Analysis working group

Harri Siirtola	University of Tampere, Tampere, Finland	**FinnGen Analysis working group**	**FinnGen Analysis working group**
Tuomo Kiiskinen	Institute for Molecular Medicine Finland (FIMM), HiLIFE, University of Helsinki, Helsinki, Finland	**FinnGen Analysis working group**	**FinnGen Analysis working group**
Jiwoo Lee	Institute for Molecular Medicine Finland (FIMM), HiLIFE, University of Helsinki, Helsinki, Finland; Broad Institute, Cambridge, MA, United States	**FinnGen Analysis working group**	**FinnGen Analysis working group**
Kristin Tsuo	Institute for Molecular Medicine Finland (FIMM), HiLIFE, University of Helsinki, Helsinki, Finland; Broad Institute, Cambridge, MA, United States	**FinnGen Analysis working group**	**FinnGen Analysis working group**
Amanda Elliott	Institute for Molecular Medicine Finland (FIMM), HiLIFE, University of Helsinki, Helsinki, Finland; Broad Institute, Cambridge, MA, USA and Massachusetts General Hospital, Boston, MA, USA	**FinnGen Analysis working group**	**FinnGen Analysis working group**
Kati Kristiansson	THL Biobank / Finnish Institute for Health and Welfare (THL), Helsinki, Finland	**FinnGen Analysis working group**	**FinnGen Analysis working group**
Mikko Arvas	Finnish Red Cross Blood Service / Finnish Hematology Registry and Clinical Biobank, Helsinki, Finland	**FinnGen Analysis working group**	**FinnGen Analysis working group**

Kati Hyvärinen	Finnish Red Cross Blood Service, Helsinki, Finland	FinnGen Analysis working group	FinnGen Analysis working group
Jarmo Ritari	Finnish Red Cross Blood Service, Helsinki, Finland	FinnGen Analysis working group	FinnGen Analysis working group
Olli Carpén	Helsinki Biobank / Helsinki University and Hospital District of Helsinki and Uusimaa, Helsinki	FinnGen Analysis working group	FinnGen Analysis working group
Johannes Kettunen	Northern Finland Biobank Borealis / University of Oulu / Northern Ostrobothnia Hospital District, Oulu, Finland	FinnGen Analysis working group	FinnGen Analysis working group
Katri Pylkäs	University of Oulu, Oulu, Finland	FinnGen Analysis working group	FinnGen Analysis working group
Eeva Sliz	University of Oulu, Oulu, Finland	FinnGen Analysis working group	FinnGen Analysis working group
Minna Karjalainen	University of Oulu, Oulu, Finland	FinnGen Analysis working group	FinnGen Analysis working group
Tuomo Mantere	Northern Finland Biobank Borealis / University of Oulu / Northern Ostrobothnia Hospital District, Oulu, Finland	FinnGen Analysis working group	FinnGen Analysis working group
Eeva Kangasniemi	Finnish Clinical Biobank Tampere / University of Tampere / Pirkanmaa Hospital District, Tampere, Finland	FinnGen Analysis working group	FinnGen Analysis working group

Sami Heikkinen	University of Eastern Finland, Kuopio, Finland	**FinnGen Analysis working group**	**FinnGen Analysis working group**
Arto Mannermaa	Biobank of Eastern Finland / University of Eastern Finland / Northern Savo Hospital District, Kuopio, Finland	**FinnGen Analysis working group**	**FinnGen Analysis working group**
Eija Laakkonen	University of Jyväskylä, Jyväskylä, Finland	**FinnGen Analysis working group**	**FinnGen Analysis working group**
Nina Pitkänen	Auria Biobank / University of Turku / Hospital District of Southwest Finland, Turku, Finland	**FinnGen Analysis working group**	**FinnGen Analysis working group**
Samuel Lessard	Translational Sciences, Sanofi R&D, Framingham, MA, USA	**FinnGen Analysis working group**	**FinnGen Analysis working group**
Clément Chatelain	Translational Sciences, Sanofi R&D, Framingham, MA, USA	**FinnGen Analysis working group**	**FinnGen Analysis working group**
Lila Kallio	Auria Biobank / University of Turku / Hospital District of Southwest Finland, Turku, Finland	**Biobank directors**	**Biobank directors**
Tiina Wahlfors	THL Biobank / Finnish Institute for Health and Welfare (THL), Helsinki, Finland	**Biobank directors**	**Biobank directors**
Jukka Partanen	Finnish Red Cross Blood Service / Finnish Hematology Registry and Clinical Biobank, Helsinki, Finland	**Biobank directors**	**Biobank directors**
Eero Punkka	Helsinki Biobank / Helsinki University and Hospital District of Helsinki and Uusimaa, Helsinki	**Biobank directors**	**Biobank directors**

Raisa Serpi	Northern Finland Biobank Borealis / University of Oulu / Northern Ostrobothnia Hospital District, Oulu, Finland	**Biobank directors**	**Biobank directors**
Sanna Siltanen	Finnish Clinical Biobank Tampere / University of Tampere / Pirkanmaa Hospital District, Tampere, Finland	**Biobank directors**	**Biobank directors**
Veli-Matti Kosma	Biobank of Eastern Finland / University of Eastern Finland / Northern Savo Hospital District, Kuopio, Finland	**Biobank directors**	**Biobank directors**
Tiina Jokela	Central Finland Biobank / University of Jyväskylä / Central Finland Health Care District, Jyväskylä, Finland	**Biobank directors**	**Biobank directors**
Anu Jalanko	Institute for Molecular Medicine Finland (FIMM), HiLIFE, University of Helsinki, Helsinki, Finland	**FinnGen Teams**	**Administration**
Auli Toivola	Institute for Molecular Medicine Finland (FIMM), HiLIFE, University of Helsinki, Helsinki, Finland	**FinnGen Teams**	**Administration**
Huei-Yi Shen	Institute for Molecular Medicine Finland (FIMM), HiLIFE, University of Helsinki, Helsinki, Finland	**FinnGen Teams**	**Administration**
Risto Kajanne	Institute for Molecular Medicine Finland (FIMM), HiLIFE, University of Helsinki, Helsinki, Finland	**FinnGen Teams**	**Administration**

Rodos Rodosthenous	Institute for Molecular Medicine Finland (FIMM), HiLIFE, University of Helsinki, Helsinki, Finland	FinnGen Teams	**Administration**
Mervi Aavikko	Institute for Molecular Medicine Finland (FIMM), HiLIFE, University of Helsinki, Helsinki, Finland	FinnGen Teams	**Administration**
Helen Cooper	Institute for Molecular Medicine Finland (FIMM), HiLIFE, University of Helsinki, Helsinki, Finland	FinnGen Teams	**Administration**
Denise Öller	Institute for Molecular Medicine Finland (FIMM), HiLIFE, University of Helsinki, Helsinki, Finland	FinnGen Teams	**Administration**
Tarja Laitinen	Institute for Molecular Medicine Finland (FIMM), HiLIFE, University of Helsinki, Helsinki, Finland	FinnGen Teams	**Administration**
Rasko Leinonen	Institute for Molecular Medicine Finland (FIMM), HiLIFE, University of Helsinki, Helsinki, Finland; European Molecular Biology Laboratory, European Bioinformatics Institute, Cambridge, UK	FinnGen Teams	**Administration**
Henna Palin	Finnish Clinical Biobank Tampere / University of Tampere / Pirkanmaa Hospital District, Tampere, Finland	FinnGen Teams	**Administration**
Malla-Maria Linna	Helsinki Biobank / Helsinki University and Hospital District of Helsinki and Uusimaa, Helsinki	FinnGen Teams	**Administration**

Mitja Kurki	Institute for Molecular Medicine Finland (FIMM), HiLIFE, University of Helsinki, Helsinki, Finland; Broad Institute, Cambridge, MA, United States	FinnGen Teams	**Analysis**
Juha Karjalainen	Institute for Molecular Medicine Finland (FIMM), HiLIFE, University of Helsinki, Helsinki, Finland	FinnGen Teams	**Analysis**
Pietro Della Briotta Parolo	Institute for Molecular Medicine Finland (FIMM), HiLIFE, University of Helsinki, Helsinki, Finland	FinnGen Teams	**Analysis**
Arto Lehisto	Institute for Molecular Medicine Finland (FIMM), HiLIFE, University of Helsinki, Helsinki, Finland	FinnGen Teams	**Analysis**
Juha Mehtonen	Institute for Molecular Medicine Finland (FIMM), HiLIFE, University of Helsinki, Helsinki, Finland	FinnGen Teams	**Analysis**
Wei Zhou	Broad Institute, Cambridge, MA, United States	FinnGen Teams	**Analysis**
Masahiro Kanai	Broad Institute, Cambridge, MA, United States	FinnGen Teams	**Analysis**
Mutaamba Maasha	Broad Institute, Cambridge, MA, United States	FinnGen Teams	**Analysis**
Zhili Zheng	Broad Institute, Cambridge, MA, United States	FinnGen Teams	**Analysis**
Hannele Laivuori	Institute for Molecular Medicine Finland (FIMM), HiLIFE, University of Helsinki, Helsinki, Finland	FinnGen Teams	**Clinical Endpoint Development**

Aki Havulinna	Institute for Molecular Medicine Finland (FIMM), HiLIFE, University of Helsinki, Helsinki, Finland; Finnish Institute for Health and Welfare (THL), Helsinki, Finland	**FinnGen Teams**	**Clinical Endpoint Development**
Susanna Lemmelä	Institute for Molecular Medicine Finland (FIMM), HiLIFE, University of Helsinki, Helsinki, Finland	**FinnGen Teams**	**Clinical Endpoint Development**
Tuomo Kiiskinen	Institute for Molecular Medicine Finland (FIMM), HiLIFE, University of Helsinki, Helsinki, Finland	**FinnGen Teams**	**Clinical Endpoint Development**
L. Elisa Lahtela	Institute for Molecular Medicine Finland (FIMM), HiLIFE, University of Helsinki, Helsinki, Finland	**FinnGen Teams**	**Clinical Endpoint Development**
Mari Kaunisto	Institute for Molecular Medicine Finland (FIMM), HiLIFE, University of Helsinki, Helsinki, Finland	**FinnGen Teams**	**Communication**
Elina Kilpeläinen	Institute for Molecular Medicine Finland (FIMM), HiLIFE, University of Helsinki, Helsinki, Finland	**FinnGen Teams**	**E-Science**
Tianduanyi Wang	Institute for Molecular Medicine Finland (FIMM), HiLIFE, University of Helsinki, Helsinki, Finland	**FinnGen Teams**	**E-Science**
Timo P. Sipilä	Institute for Molecular Medicine Finland (FIMM), HiLIFE, University of Helsinki, Helsinki, Finland	**FinnGen Teams**	**E-Science**

Oluwaseun Alexander Dada	Institute for Molecular Medicine Finland (FIMM), HiLIFE, University of Helsinki, Helsinki, Finland	**FinnGen Teams**	**E-Science**
Awaisa Ghazal	Institute for Molecular Medicine Finland (FIMM), HiLIFE, University of Helsinki, Helsinki, Finland	**FinnGen Teams**	**E-Science**
Anastasia Kytölä	Institute for Molecular Medicine Finland (FIMM), HiLIFE, University of Helsinki, Helsinki, Finland	**FinnGen Teams**	**E-Science**
Rigbe Weldatsadik	Institute for Molecular Medicine Finland (FIMM), HiLIFE, University of Helsinki, Helsinki, Finland	**FinnGen Teams**	**E-Science**
Sanni Ruotsalainen	Institute for Molecular Medicine Finland (FIMM), HiLIFE, University of Helsinki, Helsinki, Finland	**FinnGen Teams**	**E-Science**
Jaska Uimonen	Institute for Molecular Medicine Finland (FIMM), HiLIFE, University of Helsinki, Helsinki, Finland	**FinnGen Teams**	**E-Science**
Kati Donner	Institute for Molecular Medicine Finland (FIMM), HiLIFE, University of Helsinki, Helsinki, Finland	**FinnGen Teams**	**Genotyping**
Anu Loukola	Helsinki Biobank / Helsinki University and Hospital District of Helsinki and Uusimaa, Helsinki	**FinnGen Teams**	**Sample Collection Coordination**
Päivi Laiho	THL Biobank / Finnish Institute for Health and Welfare (THL), Helsinki, Finland	**FinnGen Teams**	**Sample Logistics**

Tuuli Sistonen	THL Biobank / Finnish Institute for Health and Welfare (THL), Helsinki, Finland	FinnGen Teams	**Sample Logistics**
Essi Kaiharju	THL Biobank / Finnish Institute for Health and Welfare (THL), Helsinki, Finland	FinnGen Teams	**Sample Logistics**
Markku Laukkanen	THL Biobank / Finnish Institute for Health and Welfare (THL), Helsinki, Finland	FinnGen Teams	**Sample Logistics**
Elina Järvensivu	THL Biobank / Finnish Institute for Health and Welfare (THL), Helsinki, Finland	FinnGen Teams	**Sample Logistics**
Sini Lähteenmäki	THL Biobank / Finnish Institute for Health and Welfare (THL), Helsinki, Finland	FinnGen Teams	**Sample Logistics**
Lotta Männikkö	THL Biobank / Finnish Institute for Health and Welfare (THL), Helsinki, Finland	FinnGen Teams	**Sample Logistics**
Regis Wong	THL Biobank / Finnish Institute for Health and Welfare (THL), Helsinki, Finland	FinnGen Teams	**Sample Logistics**
Auli Toivola	THL Biobank / Finnish Institute for Health and Welfare (THL), Helsinki, Finland	FinnGen Teams	**Sample Logistics**
Minna Brunfeldt	THL Biobank / Finnish Institute for Health and Welfare (THL), Helsinki, Finland	FinnGen Teams	**Registry Data Operations**
Susanna Lemmelä	Institute for Molecular Medicine Finland (FIMM), HiLIFE, University of Helsinki, Helsinki, Finland	FinnGen Teams	**Registry Data Operations**
Sami Koskelainen	THL Biobank / Finnish Institute for Health and Welfare (THL), Helsinki, Finland	FinnGen Teams	**Registry Data Operations**

Tero Hiekkalinna	THL Biobank / Finnish Institute for Health and Welfare (THL), Helsinki, Finland	FinnGen Teams	**Registry Data Operations**
Teemu Paajanen	THL Biobank / Finnish Institute for Health and Welfare (THL), Helsinki, Finland	FinnGen Teams	**Registry Data Operations**
Priit Palta	Institute for Molecular Medicine Finland (FIMM), HiLIFE, University of Helsinki, Helsinki, Finland	FinnGen Teams	**Sequencing Informatics**
Shuang Luo	Institute for Molecular Medicine Finland (FIMM), HiLIFE, University of Helsinki, Helsinki, Finland	FinnGen Teams	**Sequencing Informatics**
Mary Pat Reeve	Institute for Molecular Medicine Finland (FIMM), HiLIFE, University of Helsinki, Helsinki, Finland	FinnGen Teams	**Trajectory**
Shanmukha Sampath Padmanabhuni	Institute for Molecular Medicine Finland (FIMM), HiLIFE, University of Helsinki, Helsinki, Finland	FinnGen Teams	**Trajectory**
Marianna Niemi	University of Tampere, Tampere, Finland	FinnGen Teams	**Trajectory**
Harri Siirtola	University of Tampere, Tampere, Finland	FinnGen Teams	**Trajectory**
Javier Gracia-Tabuenca	University of Tampere, Tampere, Finland	FinnGen Teams	**Trajectory**
Mika Helminen	University of Tampere, Tampere, Finland	FinnGen Teams	**Trajectory**
Tiina Luukkaala	University of Tampere, Tampere, Finland	FinnGen Teams	**Trajectory**
Iida Vähätalo	University of Tampere, Tampere, Finland	FinnGen Teams	**Trajectory**

Iina Laak	Institute for Molecular Medicine Finland (FIMM), HiLIFE, University of Helsinki, Helsinki, Finland	**FinnGen Teams**	**Data protection officer**
Marco Hautalahti	Finnish Biobank Cooperative - FINBB	**FinnGen Teams**	**FINBB - Finnish biobank cooperative**
Johanna Mäkelä	Finnish Biobank Cooperative - FINBB	**FinnGen Teams**	**FINBB - Finnish biobank cooperative**
Saija Haapa-Paananen	Finnish Biobank Cooperative - FINBB	**FinnGen Teams**	**FINBB - Finnish biobank cooperative**
Sarah Smith	Finnish Biobank Cooperative - FINBB	**FinnGen Teams**	**FINBB - Finnish biobank cooperative**
Tom Southerington	Finnish Biobank Cooperative - FINBB	**FinnGen Teams**	**FINBB - Finnish biobank cooperative**
Meri Lähteenmäki	Finnish Biobank Cooperative - FINBB	**FinnGen Teams**	**FINBB - Finnish biobank cooperative**
